# Publisher Correction: Energy efficient perching and takeoff of a miniature rotorcraft

**DOI:** 10.1038/s44172-023-00101-3

**Published:** 2023-07-25

**Authors:** Yi-Hsuan Hsiao, Songnan Bai, Yongsen Zhou, Huaiyuan Jia, Runze Ding, Yufeng Chen, Zuankai Wang, Pakpong Chirarattananon

**Affiliations:** 1 Department of Electrical Engineering and Computer Science, Massachusetts Institute of Technology, Cambridge, MA USA; 2 Department of Biomedical Engineering, City University of Hong Kong, Tat Chee Avenue, Kowloon Tong, Hong Kong SAR, China; 3 Department of Mechanical Engineering, City University of Hong Kong, Tat Chee Avenue, Kowloon Tong, Hong Kong SAR, China; 4 Department of Mechanical Engineering, The Hong Kong Polytechnic University, Hung Hom, Kowloon, Hong Kong SAR, China

**Keywords:** Mechanical engineering, Aerospace engineering

Correction to: *Communications Engineering* 10.1038/s44172-023-00087-y, published online 13 June 2023.

In the original version of this Article, Yegor Piskarev’s contribution to the peer review process was not acknowledged. This has now been corrected in both the PDF and HTML versions of the Article.

The Peer review information section is revised to:

*Communications Engineering* thanks Alireza Ramezani, Dario Floreano, Yegor Piskarev, and the other, anonymous, reviewers for their contribution to the peer review of this work. Primary Handling Editors: Miranda Viany, Mengying Su. A peer review file is available.

