# Supplementary material for: Energy efficient perching and takeoff of a miniature rotorcraft
Source: Commun Eng. 2023 Jun 13;2:38. doi: 10.1038/s44172-023-00087-y (PMC10956013; doi:10.1038/s44172-023-00087-y)
Supplement: Supplementary file 2 — Supplementary Information [file 44172_2023_87_MOESM2_ESM.pdf]

Supplementary information for

Energy efficient perching and takeoff of a miniature rotorcraft

Yi-Hsuan Hsiao[1,2†], Songnan Bai[2†], Yongsen Zhou[3†], Huaiyuan Jia[2†], Runze Ding[2], Yufeng Chen[1], Zuankai Wang[4*], Pakpong Chirarattananon[2,3*]

[1]Department of Electrical Engineering and Computer Science, Massachusetts Institute of Technology, Cambridge, MA, USA.

[2]Department of Biomedical Engineering, City University of Hong Kong, Tat Chee Avenue, Kowloon Tong, Hong Kong SAR, China.

[3]Department of Mechanical Engineering, City University of Hong Kong, Tat Chee Avenue, Kowloon Tong, Hong Kong SAR, China.

[4]Department of Mechanical Engineering, The Hong Kong Polytechnic University, Hung Hom, Kowloon, Hong Kong SAR, China.

[†]These authors contributed equally to this work

[*]To whom correspondence should be addressed;

E-mail: zk.wang@polyu.edu.hk and pakpong.c@cityu.edu.hk

This file includes:

Supplementary Note 1: Ceiling Perching

Supplementary Note 2: Wall Perching

Supplementary Note 3: Surface Materials

Supplementary Note 4: Analysis of Thrust, Power, and Proximity Effect

Supplementary Note 5: Perching without External Feedback

Supplementary Figure 1: Mass ratio of the perching mechanisms

Supplementary Figure 2: Thrust distribution during ceiling and wall perchings

Supplementary Figure 3: Measured contact angles between water droplet and surface materials

Supplementary Figure 4: Images from a scanning electron microscope

Supplementary Figure 5: Influence of the proximity effect on thrust and input power
Supplementary Figure 6: A diagram depicting the electrical components and power of the robot
Supplementary Figure 7: Ceiling perching on dry acrylic
Supplementary Figure 8: Ceiling perching on wet acrylic
Supplementary Figure 9: Ceiling perching on dry aluminum
Supplementary Figure 10: Ceiling perching on wet aluminum
Supplementary Figure 11: Ceiling perching on dry EVA
Supplementary Figure 12: Ceiling perching on wet EVA
Supplementary Figure 13: Ceiling perching on dry wood
Supplementary Figure 14: Ceiling perching on wet wood
Supplementary Figure 15: Data from the gyroscope and accelerometer during a ceiling perching flight
Supplementary Figure 16: Preload application during the wall perching
Supplementary Figure 17: Wall perching on dry acrylic
Supplementary Figure 18: Wall perching on wet acrylic
Supplementary Figure 19: Wall perching on dry aluminum
Supplementary Figure 20: Wall perching on wet aluminum
Supplementary Figure 21: Wall perching on dry EVA
Supplementary Figure 22: Wall perching on wet EVA
Supplementary Figure 23: Wall perching on dry wood
Supplementary Figure 24: Wall perching on wet wood
Supplementary Figure 25: Experimental setup for the adhesive characterization
Supplementary Figure 26: An example of the raw measurements of the adhesion pressure test and adhesive reuseability test
Supplementary Figure 27: An example of the raw measurements of the adhesive creep resistance test
Supplementary Figure 28: Power measurement
Supplementary Figure 29: Schematic diagram of the experimental setup for power measurements

Supplementary Figure 30: Measurements and predictions for the thrust and power models
Supplementary Figure 31: Input power and onboard voltage of the proposed robot and original Crazyflie in extended hovering flights
Supplementary Figure 32: Onboard voltage of the robot in extended perching flights
Supplementary Table 1: Examples of perching aerial vehicles and their attachment mechanisms
Supplementary Table 2: Robot's physical parameters
Movie S1: Overview
Movie S2: Multi-material ceiling perching flights
Movie S3: Multi-material wall perching flights
Movie S4: Extended perching on ceiling and wall
Movie S5: Consecutive ceiling and wall perchings
Movie S6: Perchings with only onboard feedback

Supplementary Note 1
Ceiling Perching

1.1 Adhesion Pressure and Peel-off

Assuming distributed loading, the maximum tensile pressure, attributed to the normal load and bending moment, occurs at the inner edge of the pads (Figure 2b) and can be calculated according to [56, 61] as

$$\sigma_c^+ = \frac{M}{I_c}\frac{l_c}{2} + \frac{F}{A_c}, \tag{S1}$$

where M is the total external moment acting on the robot with respect to the neutral axis of the pads, F is the external force in the axial direction, $l_c/2$ is the distance from the neutral axis of the pads to the maximum tensile location, corresponding to half of the pad's length, and I_c is the moment of area of the pads, taken at its neutral axis (Figure 2b).

In this context, the moment is caused by the weight of the robot with the moment arm d_{cg}, split between two pads (Figure 1b, with $T = 0$), creating the moment $M = mgd_{cg}/2$. The axial

load in the balanced condition when $T = 0$ is $F = mg/2$. The moment of area of each adhesive pad is $I_c = w_c l_c^3/12$. Substituting these into Eq. S1 yields

$$\sigma_c^+ = \left(6\frac{d_{cg}}{l_c} + 1\right)\frac{1}{2}\frac{mg}{A_c}. \tag{S2}$$

The ratio of d_{cg} to l_c plays a pivotal role. Compared to the case where the adhesive pads were placed directly on top of the center of mass, the presented design magnifies the local maximum tensile by approximately 60 times ($d_{cg}/l_c \approx 10$, Supplementary Table 2). That is, for the surface and preload condition with maximum adhesion pressure $\sigma_{a,\max}$, the robot is able to takeoff from the ceiling through peeling off as long as $\sigma_{a,\max} < \sigma_c^+$, or

$$\sigma_{a,\max} < \left(6\frac{d_{cg}}{l_c} + 1\right)\frac{1}{2}\frac{mg}{A_c}. \tag{S3}$$

1.2 Four-Stage Ceiling Perching Scheme

The process starts with the ceiling approach (Stage I, Figure 4), with the robot initially hovering below the surface. Once commanded, the vehicle flies upwards and the ceiling contact is detected through a spike captured by the accelerator (threshold set at $1.5g$ from the baseline).

The next step concerns the application of preload and command tuning (Stage II, Figure 4). This aims to determine the minimally required collective thrust the robot needs to stay perched on a particular surface without prior knowledge of the bound of the adhesion pressure. After the ceiling detection, the vehicle maximizes the thrust generated by all four propellers for one second to preload the adhesive. The preload takes advantage of the proximity effect which amplifies the net produced thrust by over a factor of two (from 4×0.088 N to 4×0.172 N, Supplementary Figure 5c). Under this condition, the adhesive undergoes a uniform compressive load of approximately 2.2 kPa (see Supplementary Note 1). According to the adhesion tests (Figure 3a), the ceiling adhesive pair is anticipated to withstand up to 85 to 550 mN in tension, depending on the surface material.

The command tuning procedure assesses the minimally required collective thrust for the robot to stay attached to the overhang (Figure 1b) in two steps, as governed by the static conditions for equilibrium of forces (Eq. 1, $2F_c + T \geq mg$) and moments (Eq. 2, $Td_{ct} \geq mgd_{cg}$). Since the effective moment arm d_{ct} varies with the distribution of the propelling thrusts, the

parametrization of the thrust ratios is introduced as $\xi_1 = T_1/T$ and $\xi_2 = T_2/T$. With the symmetric design (Figure 1a), it is reasonable to enforce $T_4 = T_2$, leaving $T_3 = (1 - \xi_1 - 2\xi_2)T$. Furthermore, to suppress the yaw torque caused by drag attributed to the spinning propellers, the sum of thrusts from clockwise-spinning propellers ($T_2 + T_4$) and anticlockwise-spinning propellers ($T_1 + T_3$) are made equal, equivalent to imposing $\xi_2 = 1/4$, eventually resulting in $d_{ct} = d_{ct}(\xi_1)$ (defined in Note 1.3). That is, the effective distance d_{ct} is altered through the choice of a single ratio parameter ξ_1.

To evaluate the minimum moment needed (Eq. 2), d_{ct} is initially set to be the fixed distance from the adhesive pads to the geometric center of all propellers ($\xi_1 = 1/4$), close to the center of gravity of the robot. The collective thrust is steadily decreased from the maximum limit during the preload until the equilibrium of moments condition is violated, corresponding to the loss of contact between the ceiling and the pole opposite the adhesive pads or a splike in the robot's roll rate $\dot{\theta}$. This is detected through an onboard gyroscope (threshold set to 50 deg/s) and the critical ceiling perching torque $\tau_c^* = Td_{ct} = mgd_{cg}$ is recorded. Since $d_{ct}(\xi_1 = 1/4)$ and d_{cg} are approximately equal (Supplementary Note 1), the total thrust produced at τ_c^* is near mg, this means that the condition for the equilibrium of forces ($2F_c + T \geq mg$) is met during the entire process, regardless of the adhesion provided by the pads. The step yields the motor commands that approximately produce $T = mg$ without requiring prior knowledge of the actuators nor the influence of the proximity effect.

After the detection of detachment, the robot immediately re-establishes the contact with the overhang and re-applies the preload with the maximum collective thrust. It then proceeds to search for the minimum thrust $T_c^* = mg - 2F_c$ that meets the condition for the equilibrium of forces while ensuring the critical torque with a safety factor of 1.1. This is accomplished by maintaining $Td_{ct} = 1.1\tau_c^*$ and slowly and simultaneously lowering T and increasing d_{ct} proportionally via adjusting ξ_1 from $1/4$ towards 0 ($T_4 = T$ when $\xi_1 = 0$). Immediately beyond the critical state ($T < mg - 2F_c$), the adhesive detaches from the ceiling. At which point, the critical distance d_{ct}^* is obtained and the robot perches back to the ceiling with the preload re-application. The effective distance is finally selected with a conservative safety margin of 4.5 mm as $d_{ct}^* - 4.5$ mm. This ensures the robot reliably stays attached to the overhang despite some disturbances or small variations between trials. Nevertheless, in cases of strong adhesion, the

robot stays attached to the substrate even when d_{ct} is maximized at $\xi_1 = 0$. In such scenarios, the effective distance is set to $d_{ct}(\xi_1 = 0)$.

In the subsequent power conserving mode (Stage III, Figure 4), the corresponding minimum collective thrust required for safe ceiling perching is computed from $1.1\tau_c^*/(d_{ct}^* - 4.5$ mm) or $T = 1.1\tau_c^*/d_{ct}(\xi_1 = 0)$, depending on the effective distance previously obtained.

In the ideal setting, where the adhesion force to the surface is strong enough such that ξ_1 can be reduced to zero, it can be shown that the collective thrust (and power) required for the robot to stay perched is minimized to be $T = 0.78mg$ (see Supplementary Note 1). The critical adhesion pressure of the adhesive must be over 0.8 kPa to make this feasible. Based on the adhesive characterization results, all tested materials meet the demand given the anticipated preload of 2.2 kPa (blue regions in Figure 3a). In practice, the adhesive may creep under the tensile load, preventing this from being reliably achieved.

To safely transition back to flight (Stage IV, Figure 4), the robot both turns down the collective thrust and decrease d_{ct} in order to breach the equilibrium of forces or moments. The peel-off is facilitated by the robot's design and feasible as long as the maximum adhesion pressure is below the prescribed bound (Eq. S3). The large bound, thanks to the amplification by the mechanical advantage, guarantees the success of the process over a wide range of surface materials. Moreover, since the external feedback from the motion capture system was only employed for positioning the robot below the ceiling, the method can easily be adapted to remove the reliance on the external feedback as described in Supplementary Note 5 and demonstrated in Movie S6.

1.3 Thrust distribution

The location of the center of thrust (d_{ct}) is computed from τ_c/T, where τ_c is the moment produced by all four propellers respective to the adhesive pads as illustrated in Supplementary Figure 2A:

$$d_{ct} = (\sum_{i=1}^{4} T_i r_i)/(\sum_{i=1}^{4} T_i) = \frac{1}{T}\sum_{i=1}^{4} T_i r_i \tag{S4}$$

where r_i is the projected distance from the center of the ceiling adhesive pads to the ith propeller: $r_1 = r_c - r_p$, $r_2 = r_4 = r_c$, and $r_3 = r_c + r_p$ (refer to Supplementary Figure 2A and

Supplementary Table 2 for the descriptions and values of r_c and r_p). Using the definitions of ξ_1 and ξ_2 defined in the main text with $\xi_2 = 1/4$, we obtain

$$d_{ct}(\xi_1) = d_a + \frac{3}{4}d_{wb} - \xi_1 d_{wb} = r_c + r_p(\frac{1}{2} - 2\xi_1), \tag{S5}$$

with the operational range $0 \leq \xi_1 \leq 1/4$. Note that when $\xi_1 = 1/4$, d_{ct} reduces to r_c. Comparing, r_c from Supplementary Figure 2a to d_{cg} from Figure 1b, the difference between them can be neglected to simplify the analysis as the center of mass of the robot is near the geometric center of all propellers.

1.4 Preload application

During the application of preload leading to the ceiling perching, all four propellers were given the maximum duty cycle commands ($\xi_1 = 1/4$ and $d_{ct} = r_c$). The location of the collective thrust approximately coincides with the center of mass of the robot (see Supplementary Figure 2b). The magnitude of T was amplified through the proximity effect as exemplified by Supplementary Figure 5c, resulting in $T = 4 \times 0.172 = 0.69$ N. The robot remained in the equilibrium of force and moment through the normal forces acting through the support pole near propeller 3 (N_p) and two ceiling adhesive pads ($2 \times N_c$, where N_c becomes the preload). Given that both locations are approximately equidistant from the center of mass (Supplementary Figure 2b), we yield $N_p = 2N_c$ and $T - mg = N_p + 2N_c$ or $N_c = (T - mg)/4 = 93.2$ mN or 9.5 gf for $mg = 32.15$ gf. After normalized by the area $A_c = 43$ mm^2, the preload pressure is $N_c/A_c = 2.2$ kPa.

1.5 Condition for most power conservation

Based on the perching strategy outlined in the main text, we obtain the final collective thrust (without considering the safety factors)

$$T = \tau_c^*/(d_{ct}^*) = T = mgd_{cg}/d_{ct}^*. \tag{S6}$$

It can be seen that the collective thrust is minimized when the adhesive is sufficiently strong such that d_{ct}^* is minimized. This occurs when $\xi_1 = 0$ and d_{ct} becomes $r_c + r_p/2$ as captured by Eq. S5. In that situation, $T = 0.78mg$ (assuming $d_{ct} \approx r_c = 63$ mm). This means the adhesion

force per pad is $(mg - T)/2 = 0.11mg = 34.7$ mN. For $A_c = 43$ mm^2, the minimal adhesion pressure needed to yield this most efficient perching condition is 0.8 kPa. In other words, if the critical adhesion is below this value, the collective thrust must be larger than $0.78mg$. The point corresponding to the preload pressure of 2.2 kPa and critical adhesion pressure of 0.8 kPa is highlighted in Figure 3a.

Supplementary Note 2
Wall Perching

2.1 Adhesion Pressure and Peel-off

As seen in Figure 2a and Supplementary Figure 2c, the surface of the wall adhesive pads is normal to the weight of the robot when the robot is fully perched on the wall. Hence, it is the shear adhesion that supports the robot. However, some normal adhesion is required for the robot to be in the moment balance. To compute this, the stress analysis akin to Eq. S1 is considered. In this circumstance, the revolute joints render the wall pads separate entities from the rest of the vehicle. The weight of the robot is perceived by the pads as the force tangential to the surface, acting at the joint axes at the distance d_{wj} away from the surface (Figure 2d. This causes the moment about the neutral axis of each adhesive surface as $M = mgd_{wj}/2$. The maximum tensile stress is located at the top edge of the adhesive pads at the distance $l_w/2$ away from the neutral axis and the moment of area of each wall pad is $I_w = w_w l_w^3/12$. The total external axial force F, acting through the joint axes, normal to the surface, depends on the collective thrust and the normal forces on the poles. The force F is strictly negative as the sum of normal forces cannot exceed the collective thrust. Hence, the local tensile stress on the wall pads is maximized when $F = 0$. As a result, to assure the robot stays stationary on the wall, the maximum adhesion pressure with the surface must exceed

$$\sigma_{a,\max} > \frac{M}{I_w}\frac{l_w}{2} = \left(6\frac{d_{wj}}{l_w}\right)\frac{1}{2}\frac{mg}{A_w} \tag{S7}$$

where $A_w = l_w w_w$ is the surface area of each wall adhesive pad. Eq. S7 provides the lower bound of the adhesion pressure of the wet adhesive. Unlike Eq. S3, the ratio of d_{wj} to l_w is

slightly below one (Supplementary Table 2), resulting in a relatively small lower bound of the critical adhesion pressure.

To detach from the surface, the robot reduces the collective thrust such that moment attributed to the weight dominates, allowing the robot to rotate back and reduce its roll angle until θ becomes α^- (Figure 2d). At this limit, the mechanical stopper regains contact with the adhesive pads. Therefore, the main body unites with the wall pads as a single rigid entity. The condition for the adhesive peeling is again determined from the maximum local stress occurring at the upper tip of the wall adhesive pads. In this scenario, the moment caused by the robot's weight on the neutral axis of each pad is $M = mgd_{wg}\cos(\alpha^-)/2$ (Figure 2d). The axial force component, calculated from collective thrust in the normal direction to the wall, is always negative as $F = -T\sin(\alpha^-)$. Correspondingly, the bound for the maximum adhesion pressure that ensures the peel off is feasible is found (when $F, T = 0$) to be

$$\sigma_{a,\max} < \frac{M}{I_w}\frac{l_w}{2} = \left(6\frac{d_{wg}}{l_w}\cos(\alpha^-)\right)\frac{1}{2}\frac{mg}{A_w}. \tag{S8}$$

Comparing Eqs. S8 with S7, we find the ratio of the upper bound to the lower bound for the robot parameters in Supplementary Table 2 to be 11.6. The large ratio means it is easier to ensure the robot can both stay on the surface and peel off reliably.

2.2 Wall Perching procedure

The wall perching begins with the robot approaching the wall with both the wall adhesive pads in the lead as commanded by a remote operator. During flight, the weight of the wall pads keep them in the downward configuration ($\alpha \to \alpha^- = 15°$). Upon approaching the wall at a small positive roll angle θ (Figure 2d and Stage I of Figure 6a), the positive value of α^- ensures that the lower tips of the wall pads make contact with the surface (Figure 2d). Since the contact point is located below the center of mass, the normal force induces a slight roll-up torque. During the process, when the roll angle of the robot θ, is lower than α^-, the joint stoppers transmit the horizontal component of the propelling thrust on to the surface, gently preloading the adhesive against the wall (Figure 2d). The limited compressive load weakly adheres the robot to the wall. The initial contact eliminates the possible slippage as the robot perches up. Once the pads adhere to the surface, the joint angle α increases with the roll motion of the robot until the

poles reach the wall at $\alpha = \alpha^+ = 90°$. Upon reaching the wall at a small positive roll angle θ (Figure 2d and Stage I of Figure 6a), the lower tips of the adhesive pads make contact with the surface. Since the contact point is located below the center of mass, the normal force induces a slight roll-up torque. The limited compressive load weakly adheres the robot to the wall. The robot remains largely in equilibrium thanks to the horizontal component of the thrust T while the robot's weight is mostly supported by the vertical thrust component.

With the initial attachment, the translational and yaw motion of the vehicle is restricted. Meanwhile, the rotation of the robot's body is decoupled from the wall adhesive pads through the revolute joints. The dynamics of the roll angle θ in the perch-up stage (Stage II, Figure 6a) can be derived from the total applied torque (with respect to the joint axes, Figure 6b) attributed to the collective thrust and the vehicle's weight:

$$I_\theta \ddot{\theta} = T d_{jt} - mg \left(d_{jh} \cos\theta + d_{jv} \sin\theta \right), \tag{S9}$$

where I_θ is the roll moment of inertia evaluated at the joint axes, d_{jh} and d_{jv} are horizontal and vertical offsets between the center of mass and the joint axis, and d_{jt} is the effective moment arm of the collective thrust T. The critical quantity is the moment generated Td_{jt}. The effective distance d_{jt} varies with the distribution of individual propelling thrusts and their placement. Owing to the symmetry and the confined pitch kinematics introduced by the adhesive pair, it is reasonable to impose the constraint $T_2 = T_4$. For a given T, two positive ratios, ξ_1 and ξ_2 are employed to fully prescribe T_1 to T_4 as $T_1 = \xi_1 T$, $T_2 = \xi_2 T = T_4$, and $T_3 = (1 - \xi_1 - 2\xi_2)T$. As a consequence the effective moment arm becomes $d_{jt} = d_{jt}(\xi_1, \xi_2)$ (detailed in Note 2.3).

To realize the desired roll angle θ and fortify the preload, the robot switches to a wall-perching controller (see Note 2.4). As implied by Eq. S9, the customized controller is required for the robot to generate roll torque with respect to the joint axes ($\tau_\theta = Td_{jt}$), as opposed to the center of mass as in flight. Since τ_θ is a function of three quantities: T, ξ_1, and ξ_2, the demand for the roll torque commanded by the controller leaves two degrees of freedom to be manipulated. The two remaining degrees of freedom are leveraged for the robot to reinforce and maximize the preload on the adhesive pads. This is achieved by stabilizing the robot to the setpoint angle θ. In addition to satisfying the equilibrium of moments (Eq. S9 with $\ddot{\theta} = 0$), the balanced condition of the horizontal motion indicates $T \sin\theta = 2N_w$ when N_w represents the

normal force from the wall perceived by each adhesive pad (Figure 6b). Combining this with Eq. S9 when $\ddot{\theta} = 0$ to eliminate T yields

$$N_w = mg \sin\theta \frac{(d_{jh}\cos\theta + d_{jv}\sin\theta)}{2d_{jt}}. \tag{S10}$$

The result states that the preload N_w is maximized when $d_{jh}/d_{jv} = -\tan(2\theta)$ irrespective of d_{jt}. This corresponds to $\theta = \theta^* = \pi/4 + (1/2)\arctan(d_{jv}/d_{jh}) = 47°$ (Supplementary Table 2) and $N_w^* = N_w(\theta^*) = mg(d_{jv} + \sqrt{d_{jh}^2 + d_{jv}^2})/(4d_{jt})$. Moreover, since N_w is inversely proportional to d_{jt}, the preload can be further amplified through a careful selection of ξ_1 and ξ_2. This occurs when $\xi_1 = 1$ (see Supplementary Figure 16a and Note 2.3). Nevertheless, the option $\xi_1 = 1$ stipulates the propelling thrust $T_1 = \xi_1 T = 2\xi_1 N_w/\sin\theta$ which may exceed the limit of the actuator (0.12 N, Supplementary Figure 16b). To this end, the ratios $\xi_1 = \xi_2 = 1/3$ (Supplementary Figure 16a and B) were chosen to strike a balance between magnifying the preload and conforming to the propeller's thrust limit. At this setting, the motor located furthest away from the adhesive pads is off during the exertion of the preload. The preload force reaches $N_w^* = 0.30mg = 9.5$ mN. For two wall adhesive pads with the area of $A_w = 62$ mm^2 each, the average preload pressure is 1.5 kPa. However, due to the revolute joints, the adhesive pads receive non-uniform compression with the maximum pressure of 2.8 kPa at the upper tips of the pads. The adhesive characterization (Figure 3a) indicates that the critical adhesion pressure for such preload is expected be higher than the critical value of 7.4 kPa (computed from Eq. S7). That is, the preload pressure meets the minimum value required for the vehicle to stay adhered to the wall for four types of surface materials tested.

After a brief moment of the application of the preload at the roll angle θ^*, the perching setpoint is progressively moved to $90°$ to roll the robot completely against the surface. In the perched configuration ($\theta = 90°$, Stage III, Figure 6a), the weight is entirely supported by the shear adhesion. The condition for equilibrium of moments is provided by Eq. 4, that is $T \geq mgd_{jv}/d_{jt}$. To significantly reduce the collective thrust and the power consumption, the effective length d_{jt} is raised by setting $\xi_1, \xi_2 = 0$ or $T_1, T_2, T_4 = 0$, leaving only the far propeller operating and $T_3 \geq 0.05mg$ (refer to Supplementary Table. 2 for geometric parameters). The required thrust is markedly lower than the hovering state. The lower bound of the force implies minimal contact between the poles and the wall (the normal force is zero). In practice, the

generated thrust must be slightly higher to ensure the robot stays firmly in contact with the vertical surface.

Despite the adhesive being in light compression (hence, minimal creep or deformation is expected) while staying perched on the wall to conserve energy, the structural vibration induced by the spinning propeller, disturbances, or the load may still compromise the adhesion as time advances. This is alleviated through periodic reinforcement, during of which all four motors are intermittently and briefly ramped up.

To take off from the wall, the robot briefly stops all propellers and rolls down by re-engaging the perching controller. The adhesive starts to peel off according to the condition captured by Eq. S8 when the roll angle is below α^- or 15°, accelerated by a brief shut-off of all motors. Once detached from the surface, the regular flight controller takes over and the robot carries on with its mission as instructed.

Similar to the ceiling perching, the use of the motion capture system for hovering the robot near the wall before and after the perching maneuver can be readily replaced by onboard optical sensors as detailed in Supplementary Note 5 and shown in Movie S6.

2.3 Thrust distribution

The effective distance d_{jt}, measured with respect to the joint axes (Figure 1c and 6b and Supplementary Figure 2c) is the average location of all the propellers, weighted by the individual thrust. It is calculated according to

$$d_{jt} = (\sum_{i=1}^{4} T_i r_i)/(\sum_{i=1}^{4} T_i) = \frac{1}{T}\sum_{i=1}^{4} T_i r_i \tag{S11}$$

where r_i is now the projected distance from the joint axis to the ith propeller: $r_1 = r_c - w_p$, $r_2 = r_4 = r_w$, and $r_3 = r_w + r_p$ (refer to Supplementary Figure 2c and Supplementary Table 2 for the descriptions and values of r_c and r_p). Using the definitions of ξ_1 and ξ_2 defined in the main text, we have

$$d_{jt} = r_w + r_p(1 - 2\xi_1 - 2\xi_2), \tag{S12}$$

subject to $\xi_1, \xi_2 \geq 0$ and $\xi_1 + 2\xi_2 \leq 1$. For an increased normal force or preload, d_{jt} can be minimized through linear programming. The solution favors large ξ_1 or $\xi_1 = 1$, but this may

contradict the thrust limit of the propellers.

2.4 Perch-up controller

When perching up (the transitions between Stage I to II and Stage II to III in Figure 6a, excluding during the exertion of the preload), the roll torque $\tau_\theta = Td_{jt}$ was generated with $\xi_1 = \xi_2 = 1/4$, such that $T_1 = T_2 = T_3 = T_4 = T/4$ and $d_{jt} = r_c$. Provided the reference roll angle θ_d, the following proportional–integral–derivative controller with the gains k_p, k_i, k_d was implemented

$$T = \frac{1}{r_c}\left(mg(d_{jh}\cos\theta + d_{jv}\sin\theta) + I_\theta\ddot{\theta}_d + k_p(\theta_d - \theta) + k_i\int(\theta_d - \theta)\mathrm{d}t + k_d(\dot{\theta}_d - \dot{\theta})\right). \tag{S13}$$

Correspondingly, the closed-loop roll dynamics (Eq. S9) becomes

$$0 = I_\theta(\ddot{\theta}_d - \ddot{\theta}) - k_p(\theta - \theta_d) - k_i\int(\theta - \theta_d)\mathrm{d}t - k_d(\dot{\theta} - \dot{\theta}_d), \tag{S14}$$

and the stability is obtained. In practice, for slowly time-varying $\theta_d(t)$, the terms $\dot{\theta}_d$ and $\ddot{\theta}_d$ in Eq. S13 can be neglected.

During the application of preload when θ_d is set to $\theta^* = \pi/4 + (1/2)\arctan(d_{jv}/d_{jh}) = 47°$, the normal force N_w is boosted by contracting d_{jt} (Eq. S12) with $\xi_1 = 1/3$ and $\xi_2 = 1/3$. This renders $d_{jt} = r_w - r_p/3 = 59$ mm, which is shorter than r_w by $r_p/3$ or 12 mm. The control law for this period must be adjusted accordingly as

$$T = \frac{1}{r_w - r_p/3}\left(mg(d_{jh}\cos\theta + d_{jv}\sin\theta) - k_p(\theta - \theta^*) - k_i\int(\theta - \theta^*)\mathrm{d}t - k_d\dot{\theta}\right). \tag{S15}$$

2.5 Local preload pressure

Unlike the ceiling perching scenario, during the preload application of the wall perching, the pressure applies non-uniformly across the adhesive pads. To evaluate the pressure distribution, must take into account the presence of the revolute joints.

The process of preloading the adhesive pads is depicted in Supplementary Figure 16. Because of the revolute joints, only the force (not torque) is transferred to the pads at the joint

location. The force can be separated into the vertical and horizontal components. The horizontal component $T \sin\theta$ originates from the collective thrust, whereas the vertical component is the weight of the robot offsetted by the thrust component: $mg - T\cos\theta$ (see also Figure 6b).

In equilibrium at the setpoint angle $\theta = \theta^*$, it has been found that $T\sin\theta = 2N_w^* = 0.60mg$ (refer to the main text). With $\theta^* = 47°$, it follows that $T\cos\theta^* = 0.56mg$ and $mg - T\cos\theta^* = 0.44mg$. To calculate how these force elements impact the local compressive pressure perceived by the adhesive, we apply the stress analysis equation at the upper tip of the pad (where it is anticipated to peel off afterwards according to Eq. S7).

$$\sigma = \frac{M}{I_w}\frac{l_w}{2} + \frac{F}{A_w}, \tag{S16}$$

where, in this occasion, both force components contribute to the moment M and only the horizontal force is normal to the pad. This yields $M = T\sin\theta^* l_w/4 - (mg - T\cos\theta^*)d_{wj}/2$ (per pad, refer to Supplementary Figure 16 for the moment arms) and $F = T\sin\theta^*/2$. In total, Eq. S16 becomes

$$\sigma = \frac{mg}{A_w}\left(1.2 - 1.33\frac{d_{wj}}{l_w}\right), \tag{S17}$$

or $\sigma = 0.56mg/A_w = 2.8$ kPa for $l_w = 12.4$ mm and $d_{wj} = 6$ mm (Supplementary Table 2). This preload pressure is marked by a red star next to the green region in Figure3a to indicate the expected critical pressure of the adhesive at the same point (upper tip of the wall adhesive pad).

Meanwhile, the preload pressure averaged over the entire pad is found from the term F/A_w to be 1.5 kPa.

Supplementary Note 3
Surface Materials

3.1 Wettability

To characterize the wettablity of the surfaces employed for the perching experiments, we measured the static contact angles of the chosen materials using a drop shape analyzer (DSA100, KRÜSS GmbH). A surface is deemed hydrophobic or hydrophilic if its static water contact angle is over or less than $90°$. Prior to the tests, the samples were cleaned with ethanol and DI

water and dried in an oven at 80 °C. In each trial, once a droplet settled on the surface, left and right contact angles were optically recorded.

We conducted multiple tests for four investigated materials: five tests for acrylic (droplet volume: 0.55 ± 0.06 mm^3), three tests for aluminum (droplet volume: 3.0 ± 0.1 mm^3), five tests for EVA foam (droplet volume: 0.51 ± 0.06 mm^3), and five tests for wood (droplet volume: 0.60 ± 0.09 mm^3). Example photos of the measurements are shown alongside the measured angles in Figure 3.

3.2 Surface Morphology

Four substrates were analyzed by a scanning electron microscope (FEI Quanta FEG 250, FEG-SEM) at five magnification settings, with the resultant images from three representative magnification levels shown in Supplementary Figure 4. In terms of surface roughness, we found the feature size of acrylic to be lower than 1 μm. The images of aluminum show microridge-like structures that are approximately 1-5 μm apart. The images of wood suggest fibrous texture with the feature size of $5 - 20$ μm. The features are visibly less uniform. The images of EVA foam show the highest roughness, exhibiting micropores with highly inconsistent diameters of around 10-200 μm.

Supplementary Note 4
Analysis of Thrust, Power, and Proximity Effect

4.1 DAQ and Onboard Voltage Measurements

We take the data from the benchtop experiment as detailed in Materials and Methods, both with and without an overhang (111 datapoints each). Supplementary Figure 30A plots 222 voltage measurements from the flight control board V_b against the measurements from the DAQ V_i. The results display a notable discrepancy between two modes of measurements, with the onboard voltages being consistently lower than those from the DAQ. This is likely due to the inaccuracy of the onboard sensor. To account for the difference, we hypothesize that the difference is attributed to poor calibration and a possible voltage drop due to dissipative loss inside the

board. In other words, the relationship between the onboard voltage V_b and DAQ voltage V_i is anticipated to follow

$$V_i = V_b + V_o + I_i R_o, \tag{S18}$$

where I_i is the total input current, V_o is the offset, and R_o is the effective resistance to be found. To determine V_o and R_o, we carried out a linear regression to predict V_i from V_b and I_i. The best fitted values of V_o and R_o are -0.04 V and 0.20 Ω with the root-mean-square error (RMSE) of 0.2 V and the R-squared value of 0.97.

4.2 Proximity Effect

For a single propeller with radius R spinning in a quiescent air of density ρ at distance d below a flat surface, the relationship between the produced thrust and aerodynamic power as derived by momentum theory is $P_a = T_i^{2/3}/(\gamma\sqrt{2\rho\pi R^2})$, where $\gamma = \gamma(d) \geq 1$ is a coefficient capturing the proximity effect. The coefficient of the proximity effect is a monotonically increasing function of the inverse propeller-to-surface distance $1/d$ and nominally unity for a propeller infinitely far from the surface [41]. The mechanical power P_m supplied to the rotor shaft is related to P_a via an approximately constant figure of merit $\eta \leq 1$ that accounts for the efficiency of the propeller as $P_m = P_a/\eta$ or

$$P_m = \frac{1}{\gamma}\frac{T_i}{\eta}\sqrt{\frac{T_i}{2\rho\pi R^2}}. \tag{S19}$$

With Eq. S19, this implies a sizable enhancement in the thrust of a propeller operating in proximity of a surface when the mechanical input power is unchanged.

To establish the influence of the proximity effect on the developed prototype, thrust, driving voltage, and input current of the robot were measured when the vehicle was mounted on a force transducer (Supplementary Figure 28A, procedures described in Materials and Methods). During the test, all four propellers were driven by the same voltage, the collective thrust generated was directly measured. The motors were driven at various voltages and the force measurements were conducted with and without an overhang above the robot (at $d \approx 2$ mm). The measured voltage, current, and power reveal that the introduction of an overhang does not visibly affect the power consumption of the robot, but substantially amplified the collective thrust (see Supplementary Figure 5).

To determine the proximity coefficient present in Eq. S19, an analytical model based on the steady-state DC motor model was derived to evaluate the mechanical power of each motor-propeller pair from the measured current and voltages (refer to Supplementary Note 4), both in the presence and absence of the ceiling. This is accomplished by subtracting the power of flight avionics and the dissipative loss from the total power consumed by the robot. The model relates mechanical power P_m to motor's driving voltage V_m. After replacing P_m using an expression from Eq. S19, the outcome is

$$V_m = b_1\gamma^{-2/3}T_i + b_2\gamma^{-1/3}\sqrt{T_i}, \tag{S20}$$

where b_1 and b_2 are coefficients reflecting physical parameters of motors and propellers.

The plot of T_i versus V_m (Supplementary Figure 5c), from measurements with and without the overhang, provides the best-fitted value of the proximity coefficient computed using Eq. S20 as $\gamma = 2.72$ (R squared of 0.998 and RMSE of 0.04 V), implying the increase in the collective thrust close to a factor of two given the same mechanical power or motor's voltage.

4.3 Power and current distribution

Based on the experiments and measurement results described in Materials and Methods, we express the total input power consumed by the robot P_i as a product of the supplied voltage V_i and the consumed (moving average) current I_i:

$$P_i = I_i V_i. \tag{S21}$$

From the hardware perspective, the input power is distributed among components. Based on empirical evidence, P_i is the sum of three distinct parts:

$$P_i = P_{av} + 4P_{ac} + P_l, \tag{S22}$$

where P_{av} is the power of the flight avionics, P_{ac} is the actuation power delivered to one out of four motor-driven propellers P_{ac} (all motors were driven by the same command and operated under the same conditions for benchtop experiments), and P_l is the power loss caused by the circuit.

The first set of the benchtop experiments taken as described in Materials and Methods shows that the current consumed by the flight avionics was constant $I_{av} = 98.6 \pm 4.8$ mA regardless of the driving voltage V_i, meaning the power is proportional to the input voltage: $P_{av} = i_{av}V_i$ (Supplementary Figure 28c).

Based on this observation, for estimation of power consumption, we model the avionics and four motor-driven propellers to be in parallel with the voltage source as schematically depicted in Supplementary Figure 6. The current consumed by each motor I_m is given by the total current I_i after subtracting out the avionic current I_{av}: $I_i = I_{av} + 4I_m$.

4.4 Steady-state motor-propeller model

In the hardware implementation, the brushed coreless motors are not supplied by a constant voltage V_i. They are driven through electronic speed controllers that generate pulse width modulation (PWM) signals. The level of commands is governed by the duty cycle $\alpha \in [0, 1]$. Thanks to the fast PWM frequency (328 kHz, several orders of magnitude higher than the dynamics of the motor-driven propellers), the motors can be modeled as driven by a constant voltage $V_m = \alpha V_i$.

To model and esimate the current taken by each motor, we employ the 0th-order motor model:

$$V_m = I_m R_m + k\omega, \tag{S23}$$

where R_m is the total internal resistance, k is the inherent motor's electrical or torque constant, and ω is the rotor's angular rate. The inductance and inertial terms are neglected by assuming steady states.

To eliminate the dependence on ω, we incorporate the propeller's dynamics. The drag torque of a spinning propeller $\tau = c_\tau \omega^2$ [40, 41] is equated to the torque generated by the motor $\tau = kI_m$, yielding $\omega = \sqrt{I_m k / c_\tau}$. It has been shown that c_τ is approximately constant irrespective of the presence of a proximate surface [41]. Substituting the result into Eq. S23 gives

$$V_m = R_m I_m + k\sqrt{\frac{k}{c_\tau}}\sqrt{I_m} = a_1 I_m + a_2\sqrt{I_m}, \tag{S24}$$

with $a_1 = R_m$ and $a_2 = k\sqrt{k}/c_\tau$ are lumped parameters to be empirically identified.

Supplementary Figure 30b plots the measurements of I_m and V_m (taken as αV_i) obtained at various duty cycle signals. The best-fitted line represents the predictions of V_m from the measured current I_m based on the best-fitted coefficients $a_1 = 1.42\ \Omega$, $a_2 = 2.70\ \mathrm{VA}^{-0.5}$, with the R-squared value of 0.995. The RMSE is 0.06 V. It can also be seen that the datapoints from with and without ceiling measurements collapse, supporting the assumption that c_τ is approximately constant. In other words, the relation between I_m and V_m described by Eq. S24 applies generally, independent of the proximity effect.

To yield $V_m = \alpha V_i$ from the onboard voltage V_b, we must incorporate Eq. S18 by writing $V_m = \alpha V_i = \alpha(V_b + V_o + I_i R_o)$. Since $I_i = I_{av} + 4I_m$, we obtain

$$V_m = \alpha \left(V_b + V_o + (I_{av} + 4I_m)R_o\right). \tag{S25}$$

Equating this with Eq. S24 to eliminate V_m produces

$$\alpha \left(V_b + V_o + I_{av}R_o\right) + (4\alpha R_o - a_1)I_m = a_2\sqrt{I_m}. \tag{S26}$$

By squaring Eq. S26 and algebraically manipulating the result (treating the equation as a quadratic equation of I_m), we obtain I_m as

$$I_m = \frac{\left(2\alpha \left(V_b + V_o + I_{av}R_o\right)\left(a_1 - 4\alpha R_o\right) + a_2^2\right) - \sqrt{a_2^4 + 4\left(V_b + V_o + I_{av}R_o\right)\left(a_1 - 4\alpha R_o\right)a_2^2}}{2(a_1 - 4\alpha R_o)^2}. \tag{S27}$$

This is essential as a current sensor is unavailable on the onboard avionics. The presented formula allows I_m to be estimated from pre-determined model coefficients a_1, a_2 and flight logs containing the motor duty cycle commands and the onboard voltage (V_b).

To validate the predictions of the motor current (Eq. S27), Supplementary Figure 30c shows the measured individual motor current alongside the model predictions using the fitted coefficients for a range of duty cycle commands and supplied voltage (as measured by the flight board). The R squared value and RMSE were found to be 0.990 and 23 mA, indicating the fidelity of the model.

4.5 Modeling of power consumption

The expression of I_m above can be used to predict the total power consumption of the robot from V_b and α from $P_i = I_i V_i$ or

$$P_i = (I_{av} + 4I_m)\left(V_b + V_o + (I_{av} + 4I_m)R_o\right). \tag{S28}$$

Combined with I_m from Eq. S27, the outcome allows the total power to be estimated from the onboard voltage measurements V_b and motor commands α. The total power measured from the benchtop experiment is plotted against the duty cycle commands and onboard voltage, overlaid by the model predictions, as shown in Supplementary Figure 30d. The R squared value and RMSE were found to be 0.983 and 0.39 W, suggesting that the model can be used to accurately estimate the power dissipated in flight when only onboard measurements are avaialble.

For actual flights, the duty cycle and current of all motors differ. The total power consumption can be calculated based on Eq. S28 after a simple modification,

$$P_i = (I_{av} + \sum_{k=1}^{4} I_{m,k})\left(V_b + V_o + (I_{av} + \sum_{k=1}^{4} I_{m,k})R_o\right), \tag{S29}$$

where $I_{m,k}$ refers to the current belonging to the kth motor.

4.5.1 Power loss

To account for the total power consumption, it vital to include all power dissipating components outlined in Eq. S22. As depicted by Supplementary Figure 6, in addition to the avionic power, the power delivered to the robot is supplied to four motors, but with some loss captured by P_l.

The power loss P_l is originated by the electronic design of the flight controller board. From the supplied voltage V_i and total current I_i, the leftover current, $4I_m = I_i - I_{av}$ is consumed by the motors. However, the actual power delivered to the motor $4V_m I_m = 4\alpha V_i I_m$ is only a fraction of the remaining power $4V_i I_m$, incurring the loss of $P_l = 4(1-\alpha)V_i I_m$. The loss is greater when the robot operates at low duty cycle commands, reflecting the deficiency in the power electronics. It is foreseeable that a flight control board with improved power management would allow the robot to conserve even more power while perching, during of which the duty cycle commands are low.

4.6 Motor voltage, mechanical power and propelling thrust

To verify and quantify the thrust enhancement induced by the proximity effect as predicted by MT (Eq. S19), we focus on the mechanical power, measured thrust forces, and motor voltage. To relate the result from MT (Eq. S19) to the motor's model (Eq. S23), we use the fact that $P_m = \tau\omega = c_\tau\omega^3$. With the earlier finding that $\omega = \sqrt{I_m k/c_\tau}$, we may write $P_m = I_m k\sqrt{I_m k/c_\tau}$ or $I_m = P_m^{\frac{2}{3}} c_\tau^{\frac{1}{3}}/k$. Substitute the outcome back into the motor's model (Eq. S23), we acquire

$$V_m = \frac{c_\tau^{\frac{1}{3}} R_m}{k} P_m^{\frac{2}{3}} + \frac{k}{c_\tau^{\frac{1}{3}}} P_m^{\frac{1}{3}}. \quad \text{(S30)}$$

Subsequently, substituting mechanical power expressed in Eq. S19 into Eq. S30 yields

$$V_m = (c_\tau^{1/3}/k) R_m (\eta^{-1}\gamma^{-1}\frac{1}{\sqrt{2\rho\pi R^2}})^{2/3} T + (k/c_\tau^{1/3})(\eta^{-1}\gamma^{-1}\frac{1}{\sqrt{2\rho\pi R^2}})^{1/3}\sqrt{T}. \quad \text{(S31)}$$

When constant parameters are lumped together, Eq. S31 reduces to $V_m = b_1\gamma^{-2/3}T + b_2\gamma^{-1/3}\sqrt{T}$ where b_1 and b_2 are to be experimentally determined. The fitted result presented in the main text (Supplementary Figure 5c) was obtained with the best-fitted coefficients: $b_1 = 24.18$ V/N, $b_2 = 4.13$ V/N$^{0.5}$, and $\gamma = 2.72$.

Supplementary Note 5

Perching without External Feedback

In the previous ceiling and wall perching experiments, motion capture cameras were used for indoor flights. The position feedback provided by the motion capture system was used to hover the robot steadily underneath the ceiling or in front of the wall. During the approach and the perching, only onboard feedback from the inertial sensor was employed to detect the surface and determine the current state of the robot. The position information from the motion capture system was then used again to stabilize the position of the robot after the detachment from the ceiling or wall.

To demonstrate that the developed mechanisms and maneuvers can be transferred to uses outside the laboratory environment, we eliminated the need for position feedback from the motion capture system by employing additional onboard optical sensors. The robot was equiped

with a set of time-of-flight and optical flow sensor (Bitcraze, Flow deck v2) in a 1.6 g package. The deck enables simple positioning through the altitude measurement provided by the time-of-flight sensor and measurements of the horizontal velocities from the optical flow. Without markers for the motion capture system, the total mass of the robot was 31.9 g, almost identical to the previous prototype.

With the ability to hover steadily (by minimizing lateral velocities and maintaining the altitude), the robot could be controlled by a human operator to stay stably under a horizontal surface or in front of a vertical surface. The same perching routines previously elaborated permitted the robot to approach the surfaces and completed the perching maneuvers without the motion capture system as demonstrated in Movie S6.

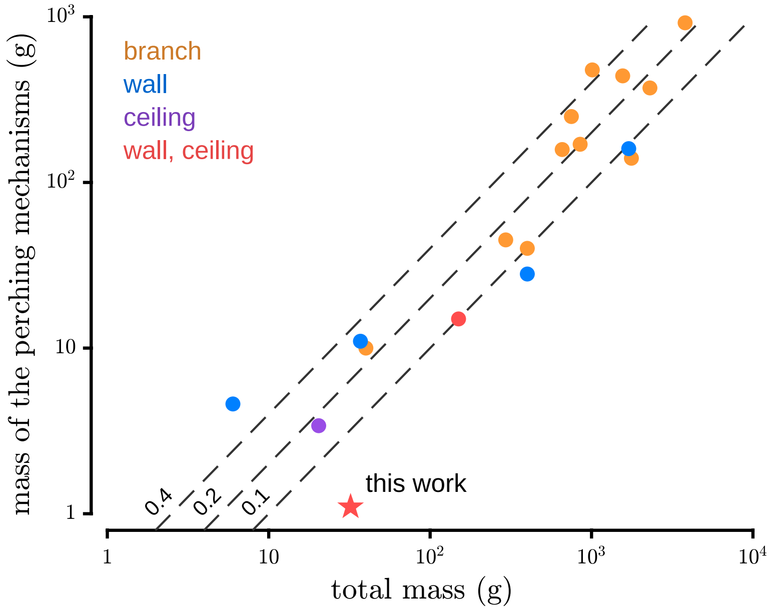

Supplementary Figure 1: Mass ratio of the perching mechanisms (refer to Supplementary Table 1 for itemized data and sources). The plot displays the mass of the mechanisms against the total mass of the robots in logarithmic scales, categorized by the perching ability. The dashed lines represent the weight ratios of the mechanisms with respect to the total mass.

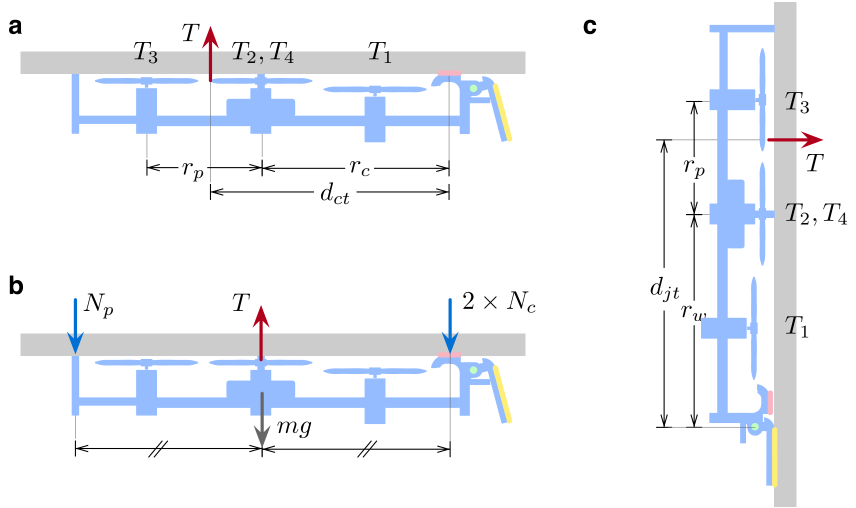

Supplementary Figure 2: Thrust distribution during ceiling and wall perchings. (a) The location of the collective thrust with respect to the neutral axis of the wall adhesive pads. (b) Force distribution during the preload application. (c) The location of the collective thrust with respect to the joint axes during wall perching.

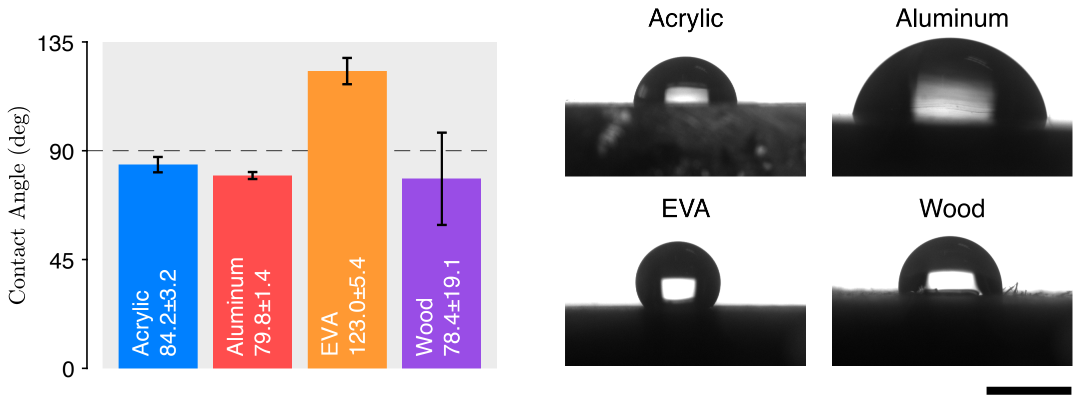

Supplementary Figure 3: Measured contact angles between water droplet and surface materials. (left) Bar plots of the measured contact angles, showing the average from 3-5 samples and the error bars indicating one standard deviation. (right) Optical images of the static droplets on four tested materials, with a scale bar of 1 mm.

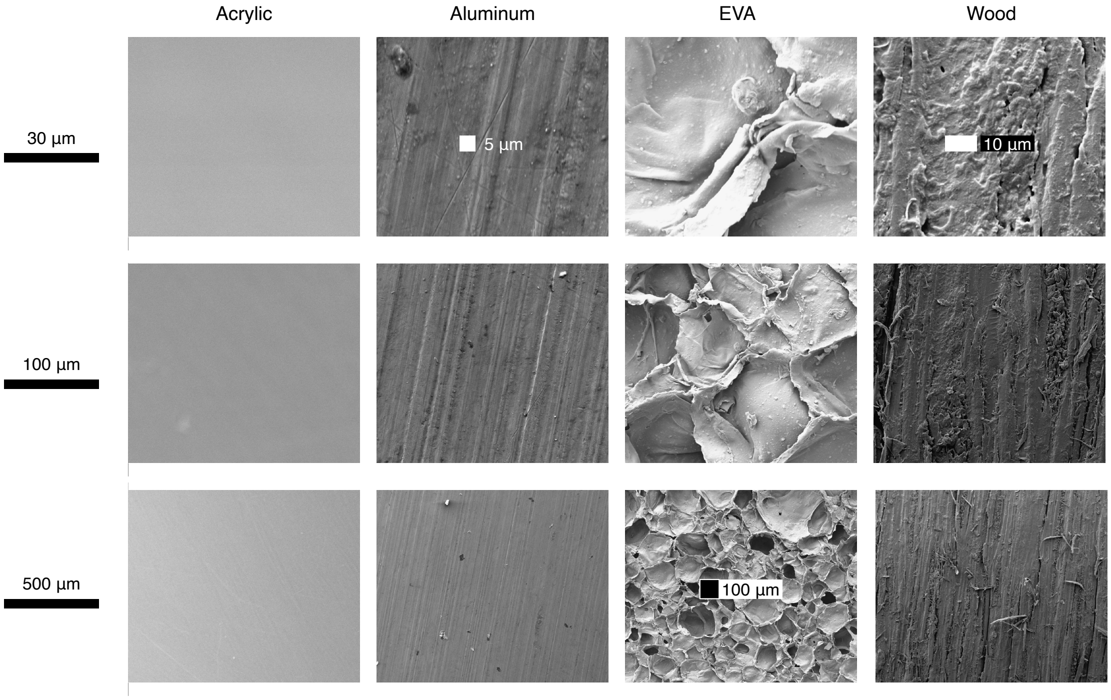

Supplementary Figure 4: Images from a scanning electron microscope.

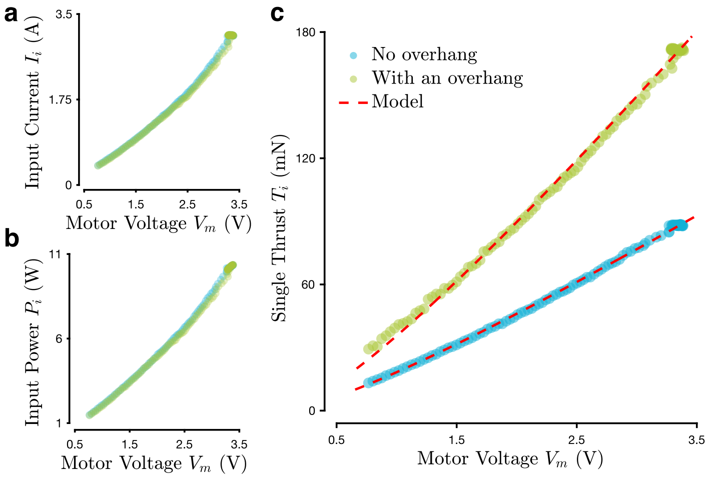

Supplementary Figure 5: Influence of the proximity effect on thrust and input power. (a) The measurements show that the total current consumed by the vehicle depends on the motor's voltage, regardless whether the ceiling is present or not. (b) The total power consumption displays a similar trend. (c) Unlike the input power, the propelling thrust is markedly impact by the overhang. The fitted model suggests the proximity coefficient of $\gamma = 2.72$.

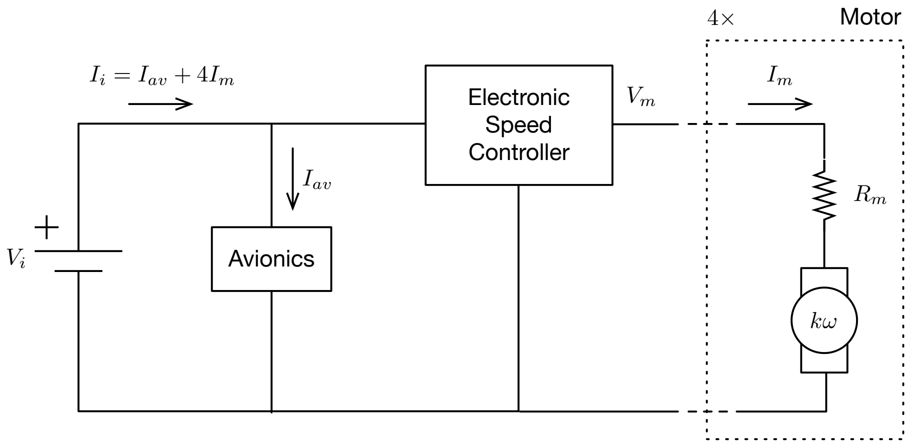

Supplementary Figure 6: A diagram depicting the electrical components and power of the robot. The avionics consume a fixed amount of current I_{av} regardless of the supplied voltage V_i. The electronic speed controller steps down the voltage according to the motor commands by conserving the current, resulting in some power loss when V_m is below V_i.

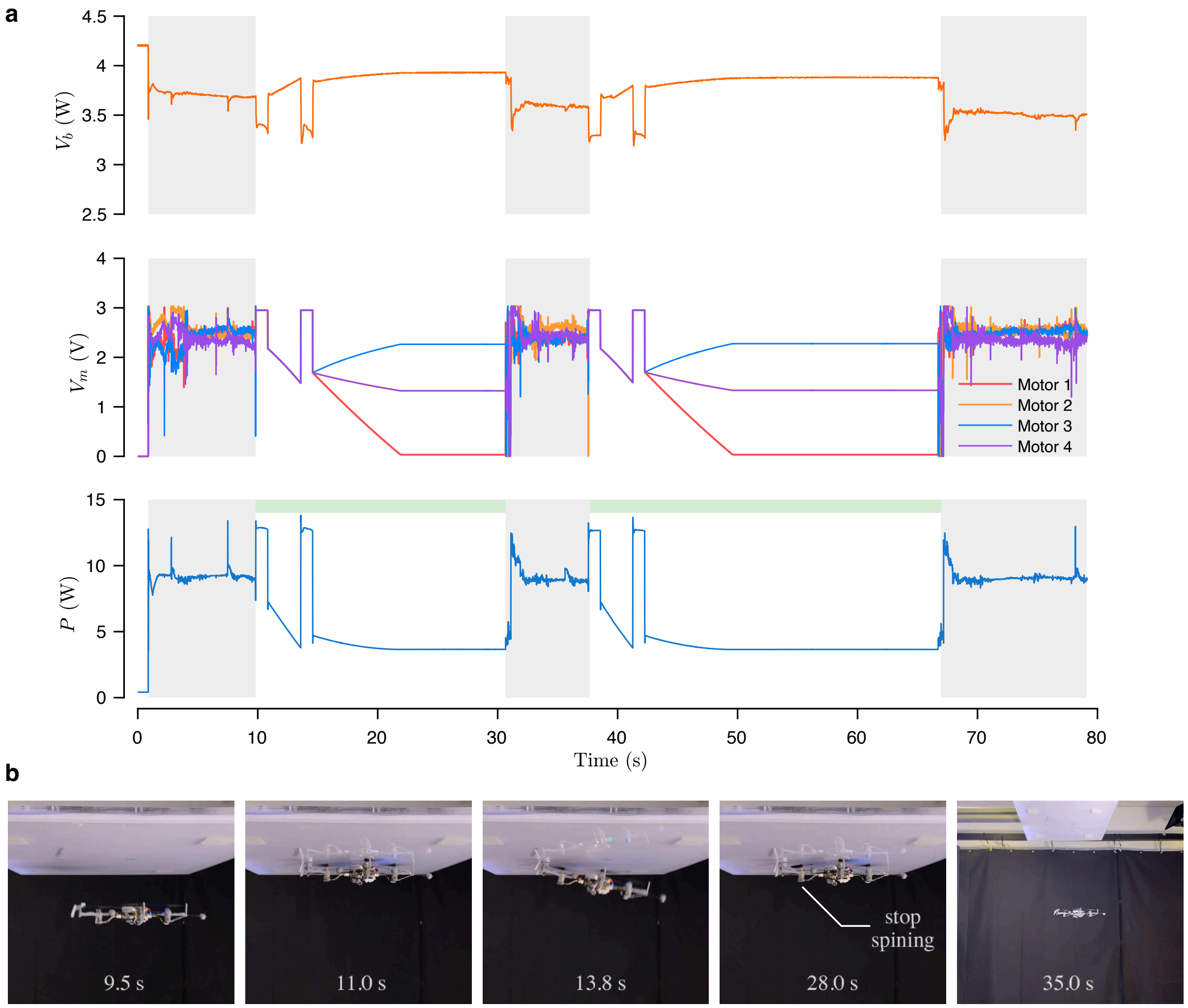

Supplementary Figure 7: Ceiling perching on dry acrylic. (a) Plots of flight data taken from the consecutive ceiling perching experiment. (b) A sequence of images showing different stages of the ceiling perching from approaching to taking off.

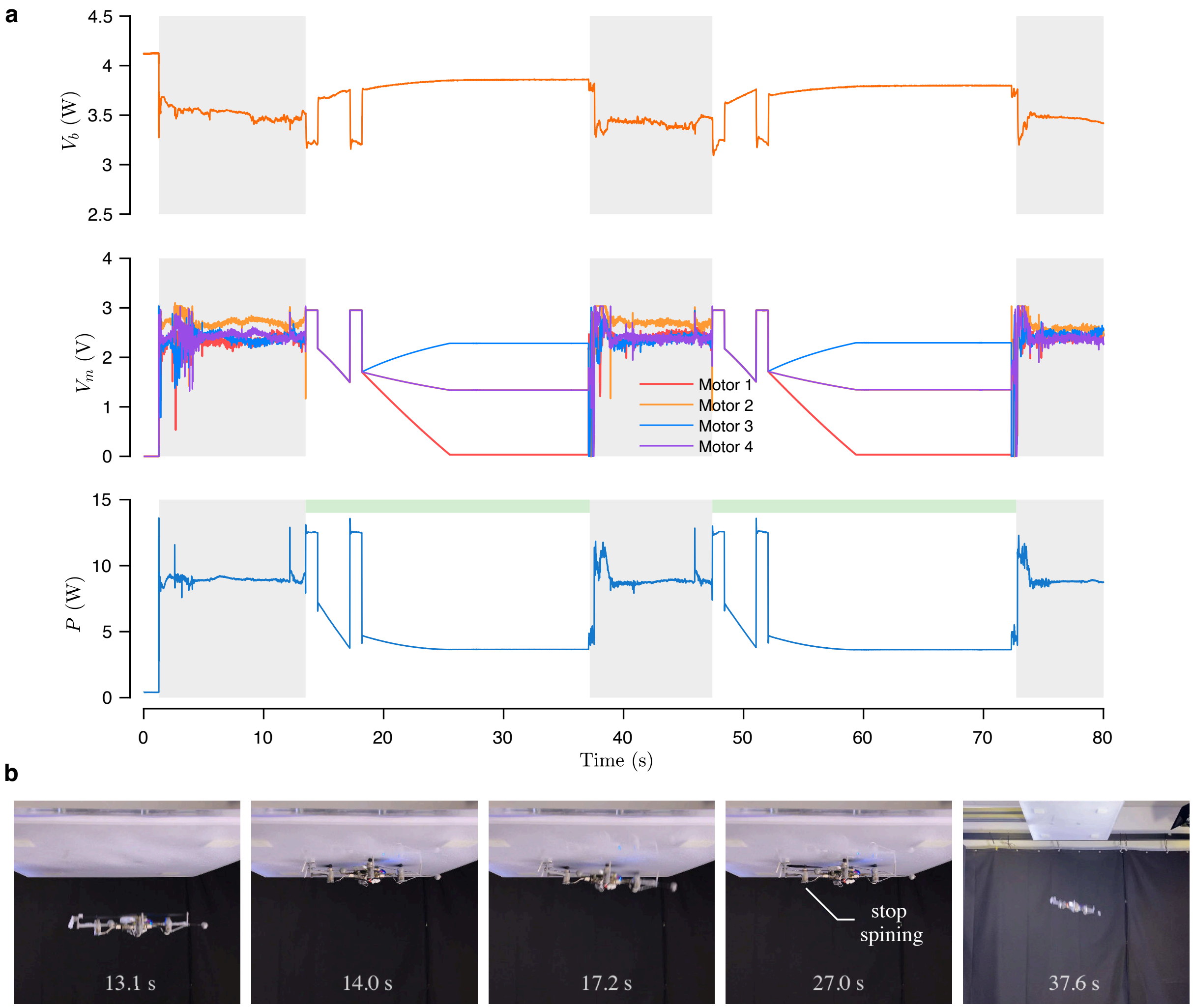

Supplementary Figure 8: Ceiling perching on wet acrylic. (a) Plots of flight data taken from the consecutive ceiling perching experiment. (b) A sequence of images showing different stages of the ceiling perching from approaching to taking off.

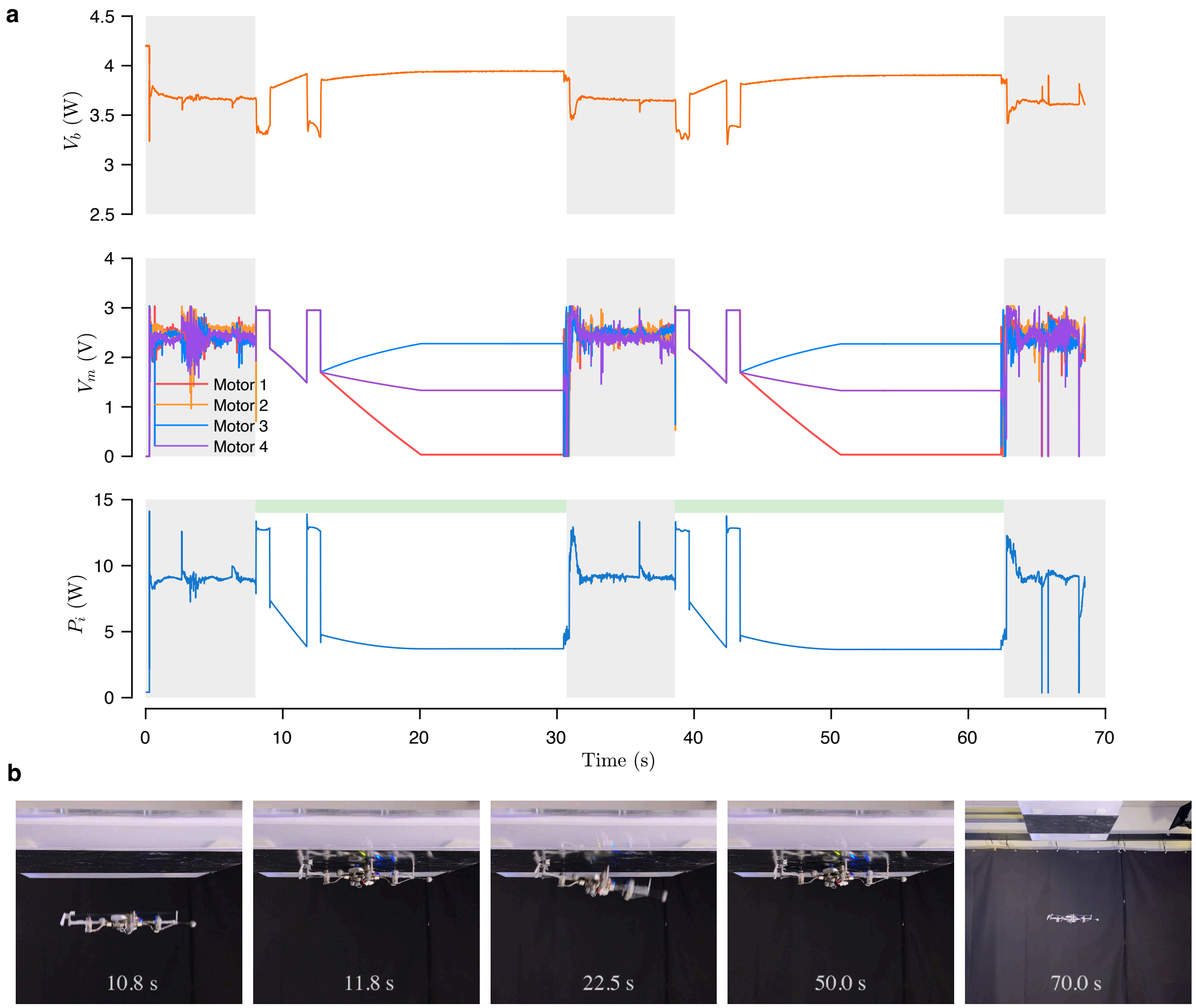

Supplementary Figure 9: Ceiling perching on dry aluminum. (a) Plots of flight data taken from the consecutive ceiling perching experiment. (b) A sequence of images showing different stages of the ceiling perching from approaching to taking off.

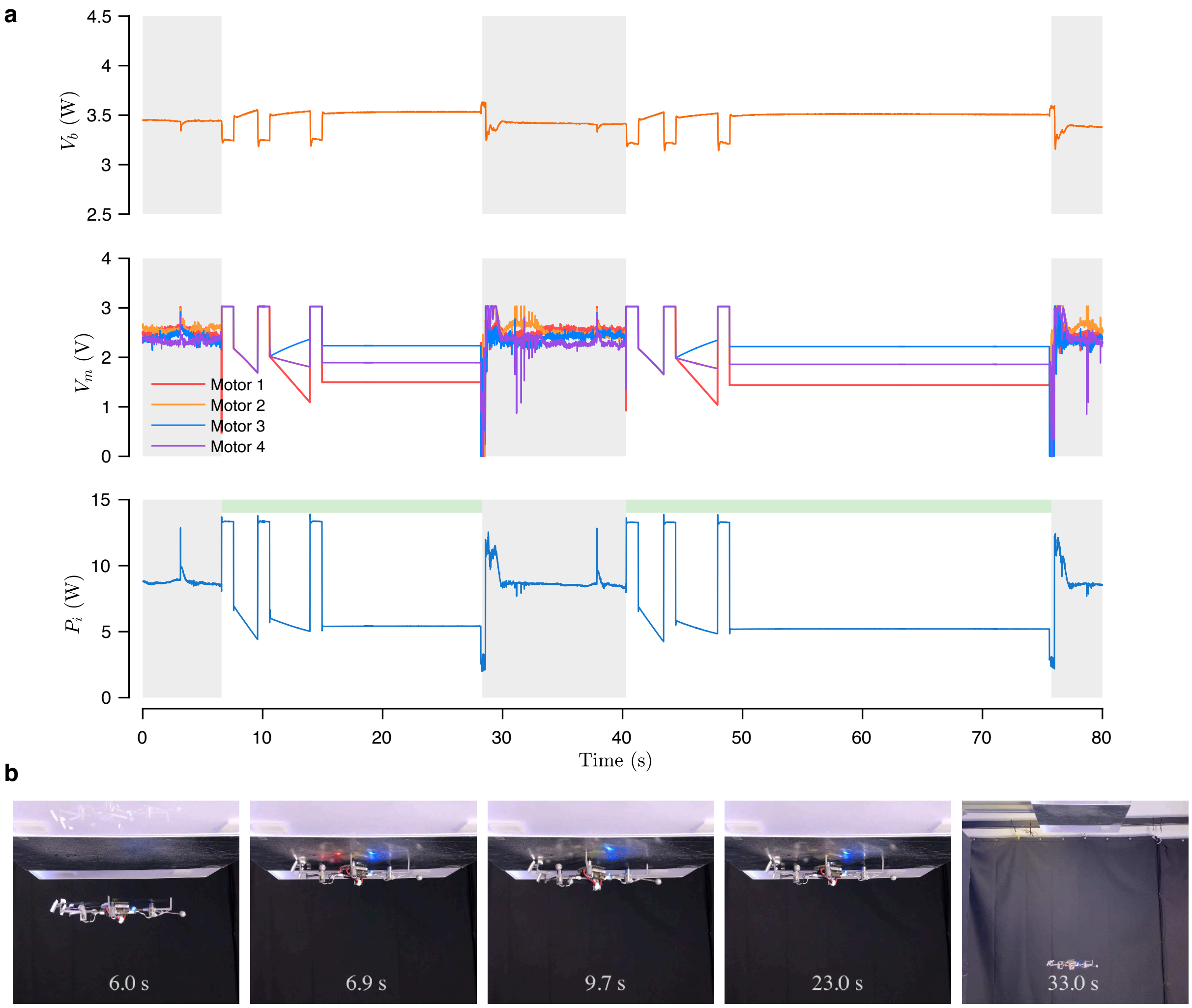

Supplementary Figure 10: Ceiling perching on wet aluminum. (a) Plots of flight data taken from the consecutive ceiling perching experiment. (b) A sequence of images showing different stages of the ceiling perching from approaching to taking off.

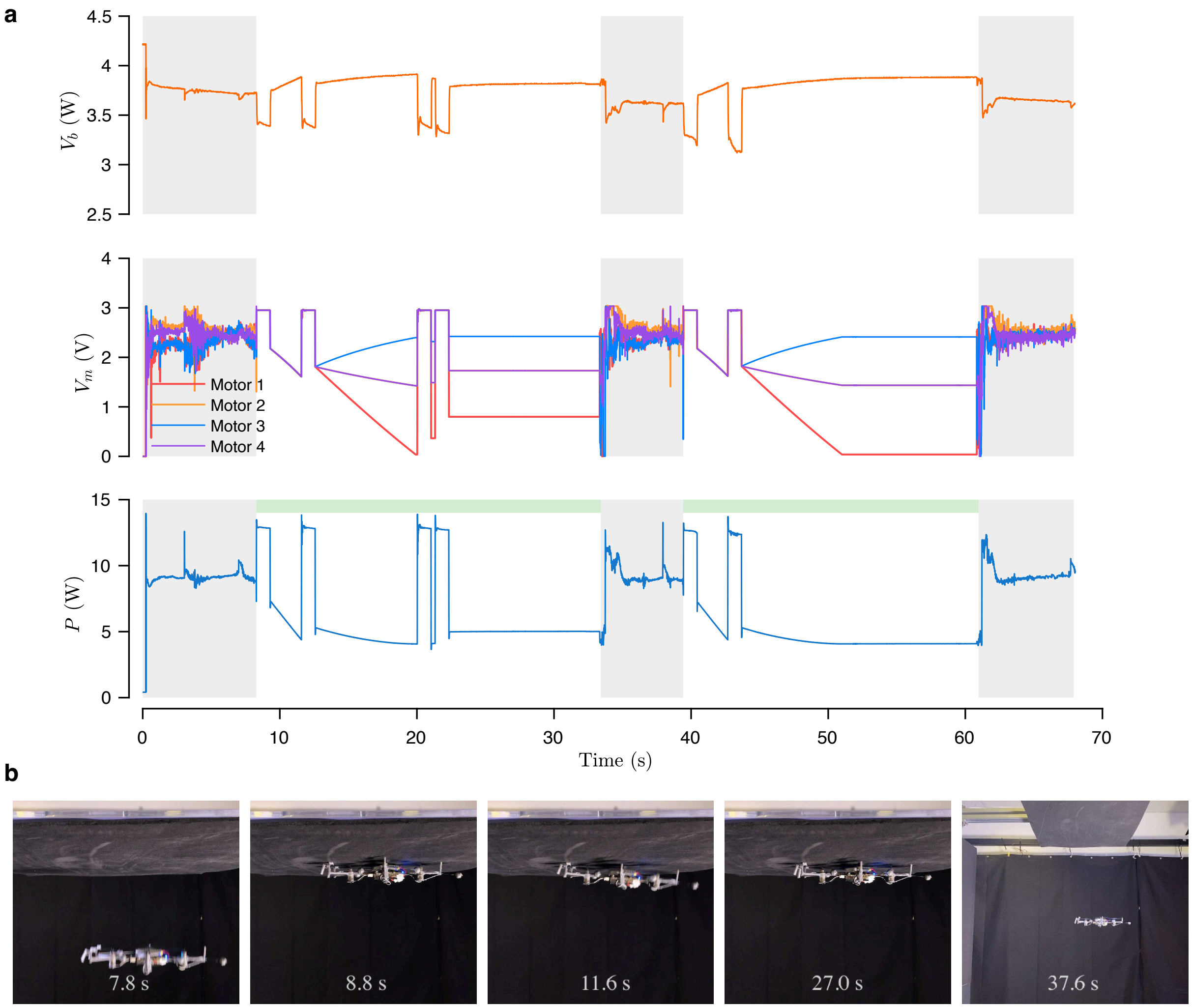

Supplementary Figure 11: Ceiling perching on dry EVA. (a) Plots of flight data taken from the consecutive ceiling perching experiment. (b) A sequence of images showing different stages of the ceiling perching from approaching to taking off.

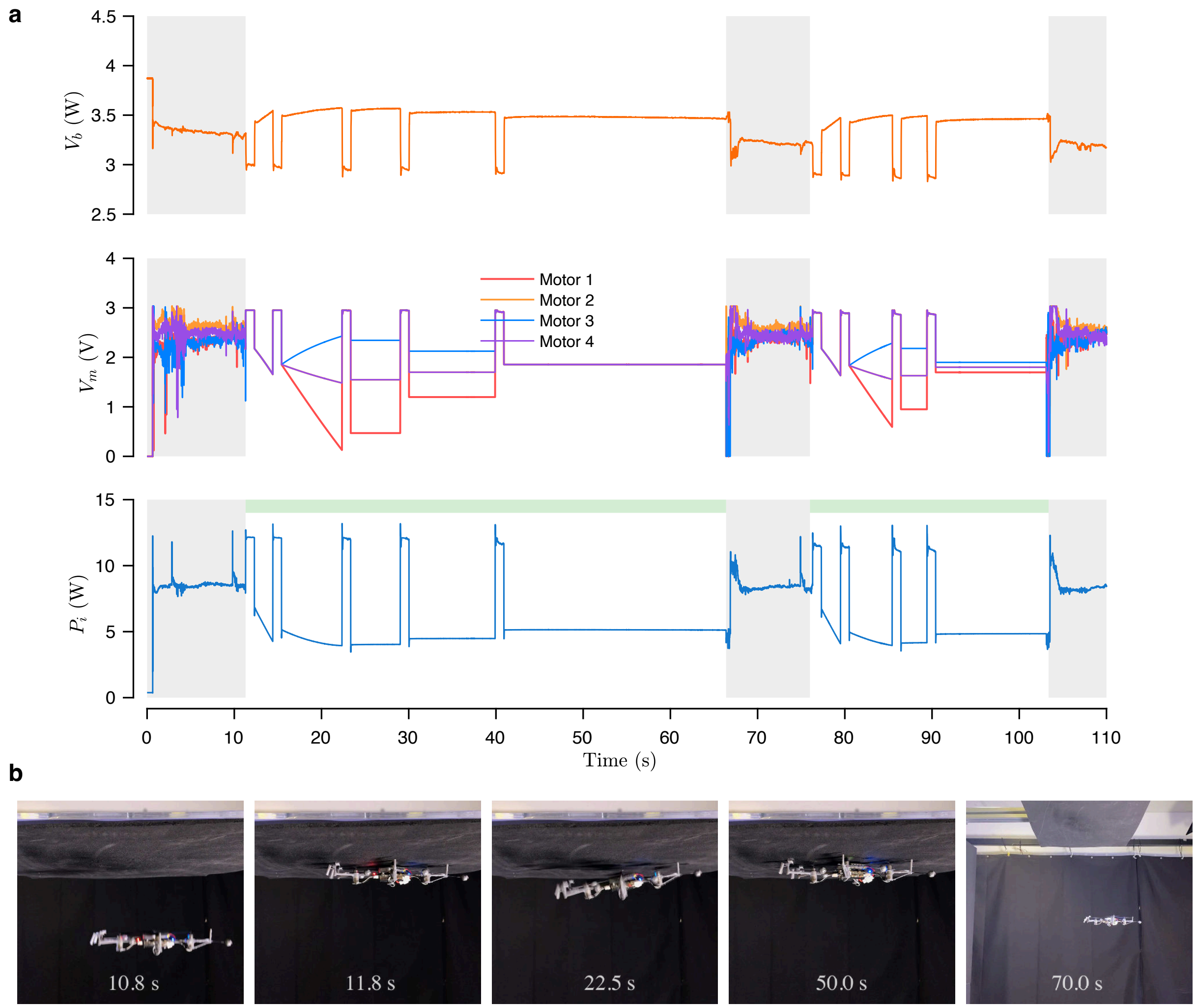

Supplementary Figure 12: Ceiling perching on wet EVA. (a) Plots of flight data taken from the consecutive ceiling perching experiment. (b) A sequence of images showing different stages of the ceiling perching from approaching to taking off.

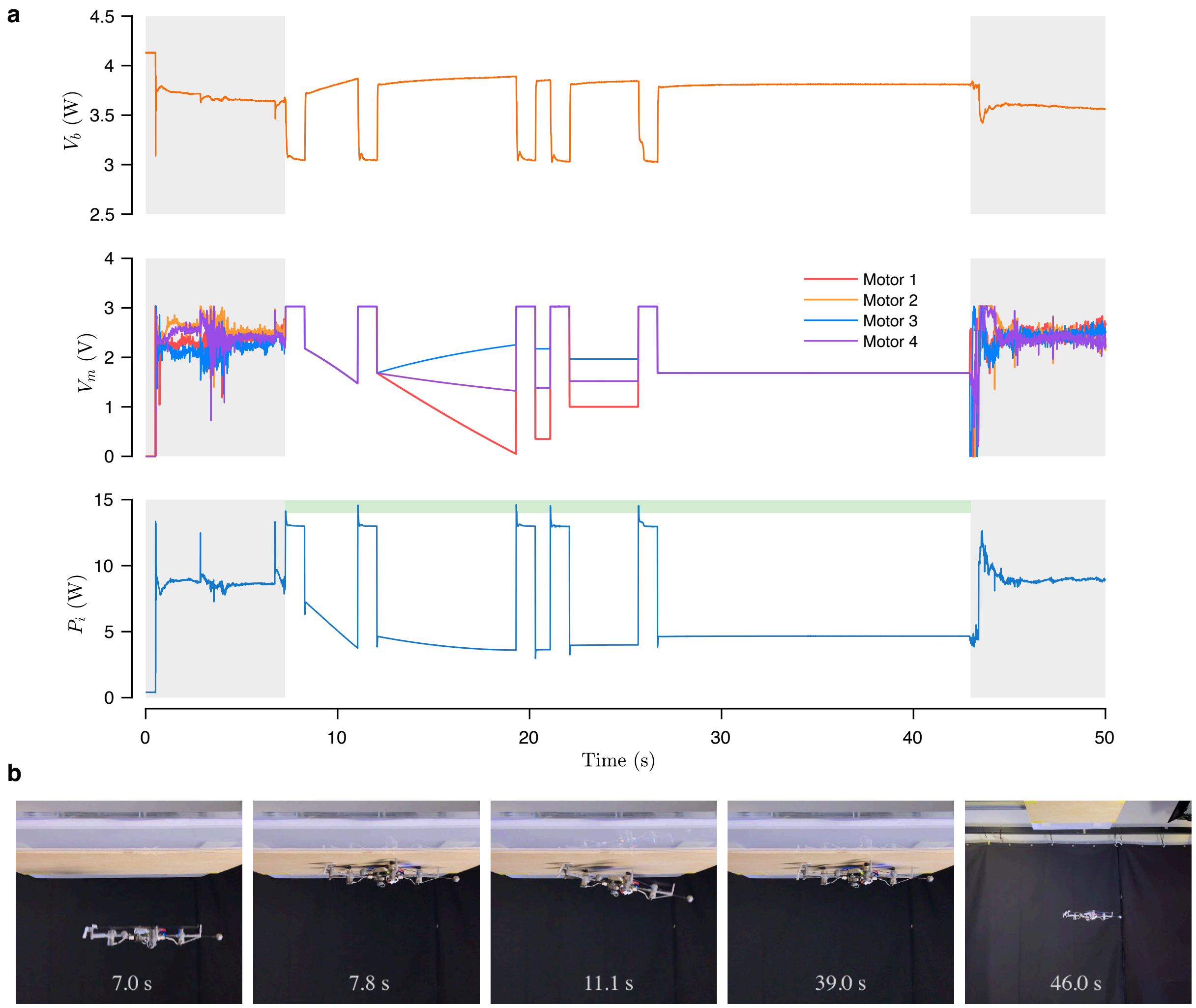

Supplementary Figure 13: Ceiling perching on dry wood. (a) Plots of flight data taken from the consecutive ceiling perching experiment. (b) A sequence of images showing different stages of the ceiling perching from approaching to taking off.

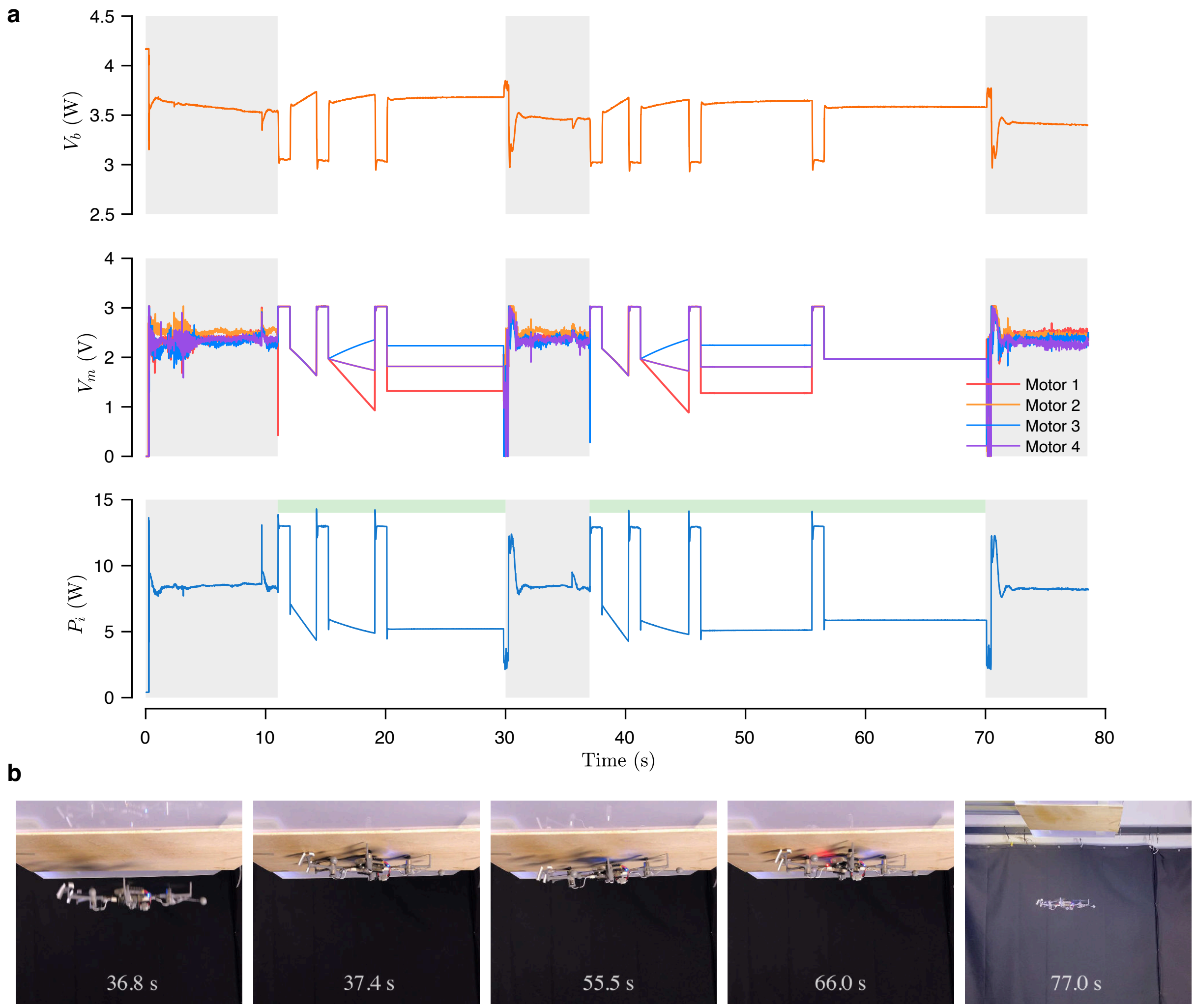

Supplementary Figure 14: Ceiling perching on wet wood. (a) Plots of flight data taken from the consecutive ceiling perching experiment. (b) A sequence of images showing different stages of the ceiling perching from approaching to taking off.

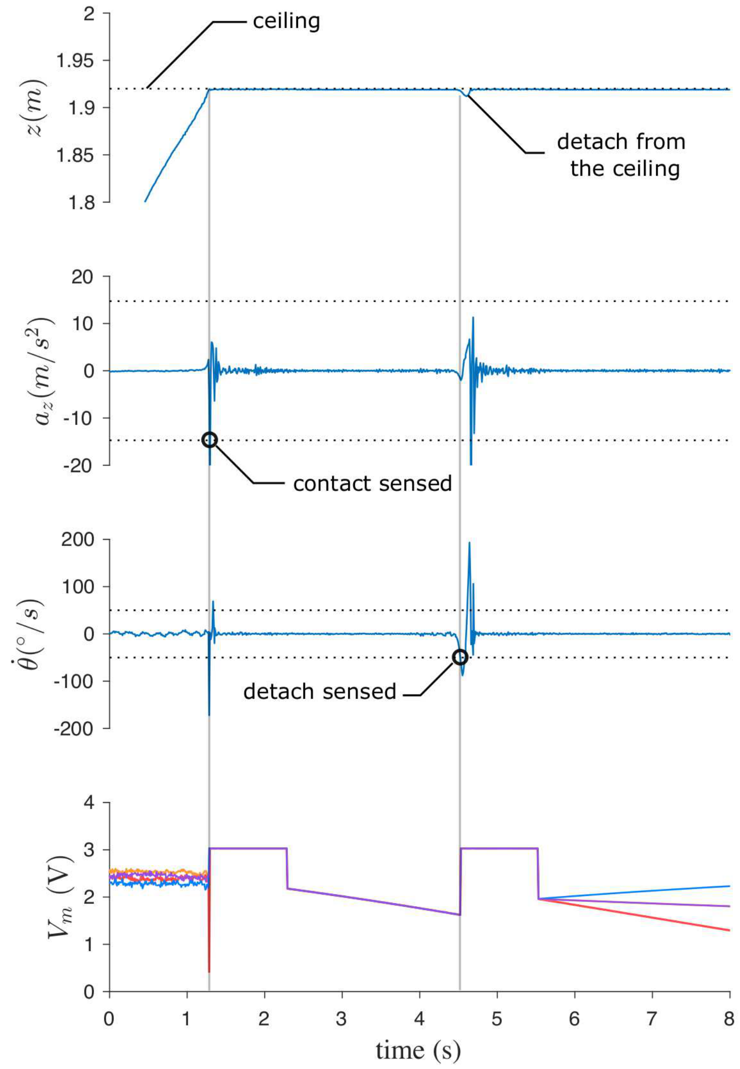

Supplementary Figure 15: Data from the gyroscope and accelerometer during a ceiling perching flight. The robot sensed the contact with the ceiling through the accelerometer. The gyroscope reading was used to detect the detachment with the ceiling. The thresholds for detection are marked by the doted lines.

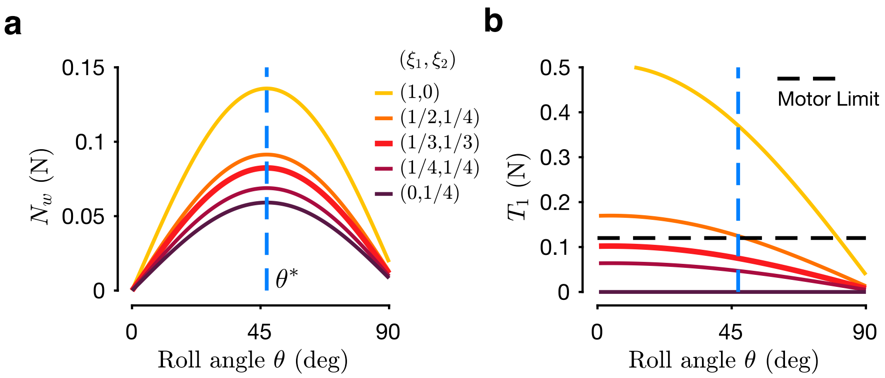

Supplementary Figure 16: Preload application during the wall perching. Due to the revolute joint, only forces are transferred to the wall adhesive pads through the joints. (a) The normal or preload force on each wall adhesive pad at different motor commands and roll angle. (b) The thrust nominally required from propeller 1 for the robot to keep the roll angle constant during the application of preload.

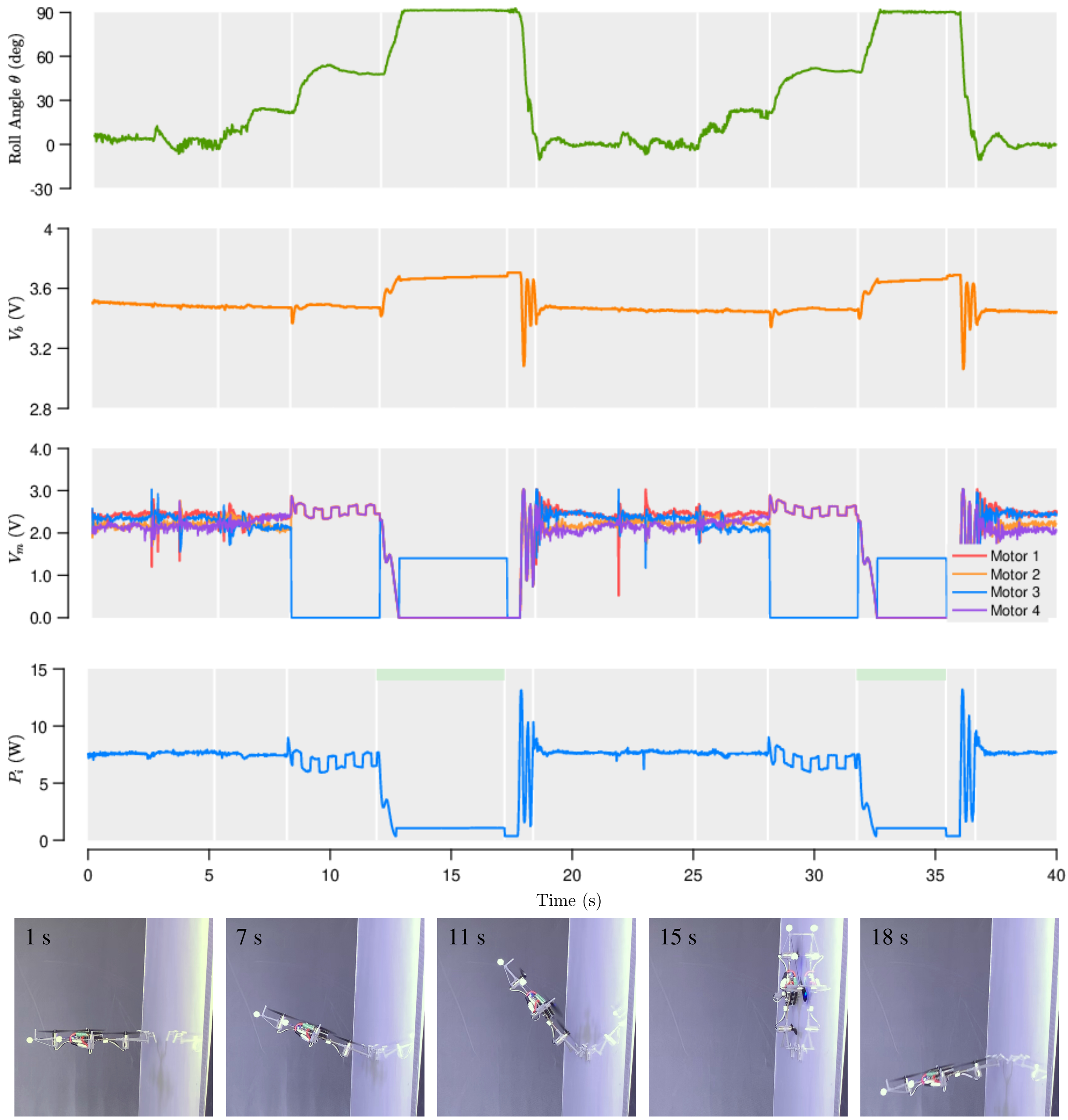

Supplementary Figure 17: Wall perching on dry acrylic. (Top) Plots of flight data taken from the consecutive wall perching experiment. (Bottom) A sequence of images showing different stages of the wall perching from approaching to taking off.

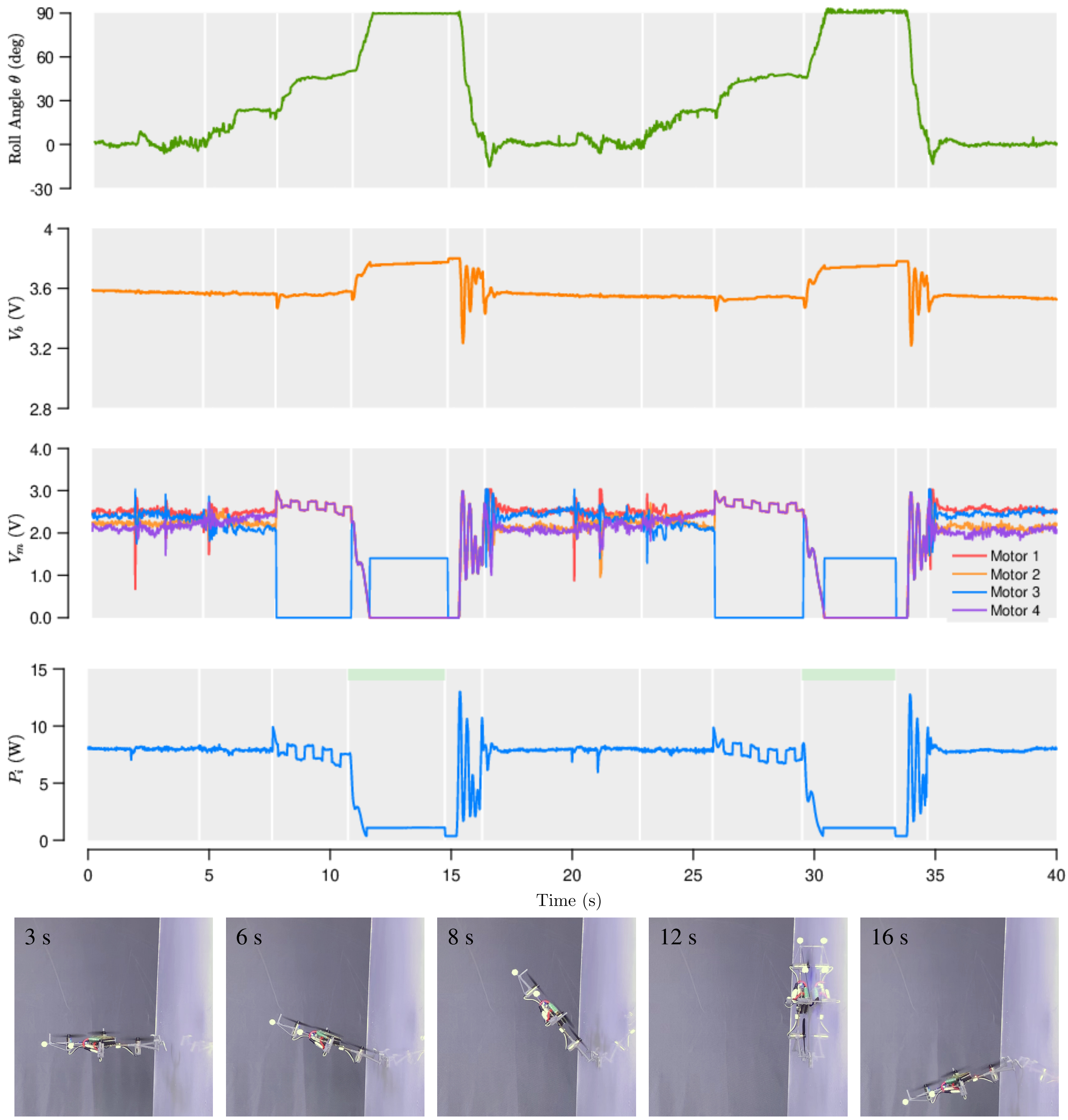

Supplementary Figure 18: Wall perching on wet acrylic. (Top) Plots of flight data taken from the consecutive wall perching experiment. (Bottom) A sequence of images showing different stages of the wall perching from approaching to taking off.

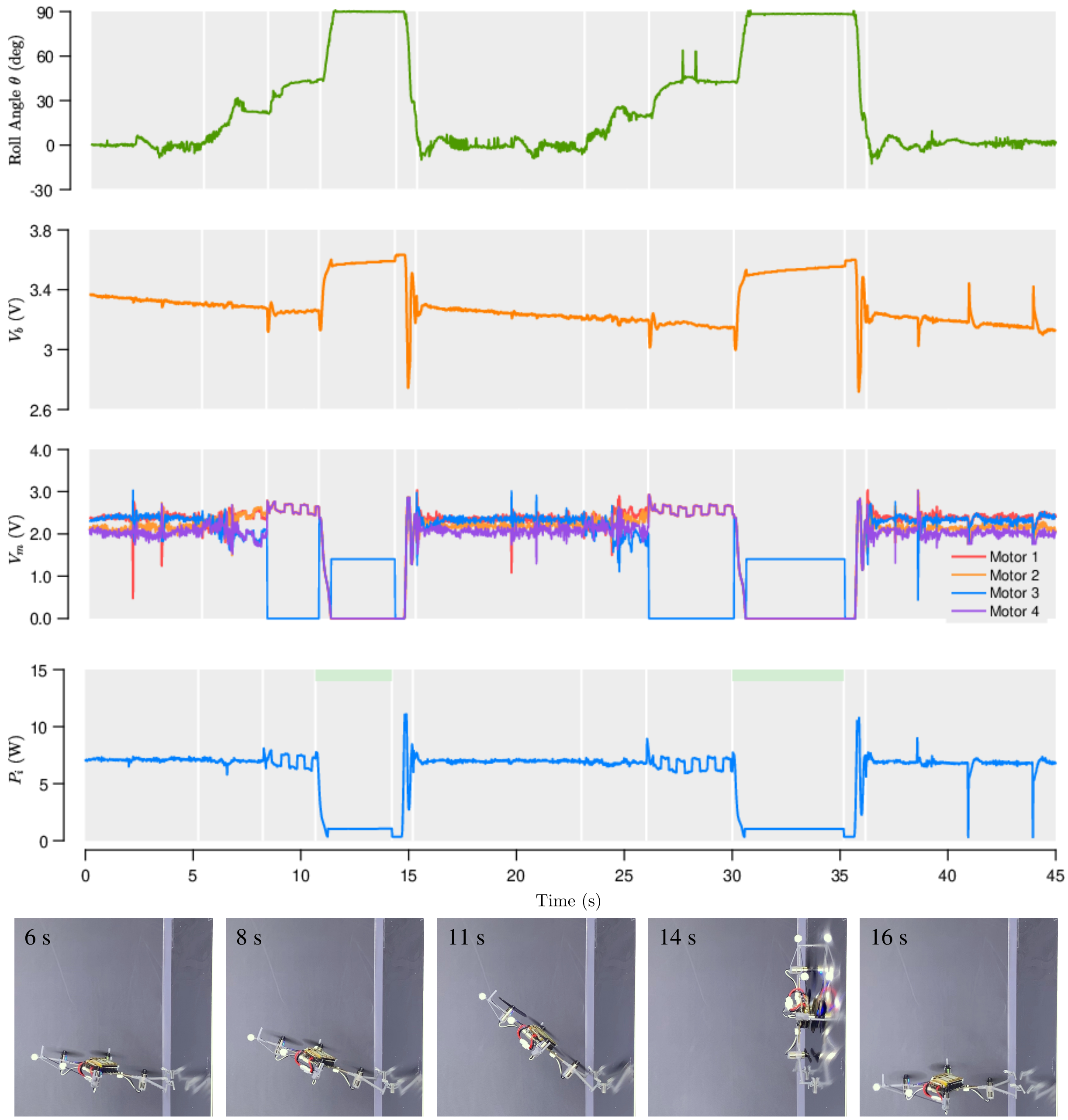

Supplementary Figure 19: Wall perching on dry aluminum. (Top) Plots of flight data taken from the consecutive wall perching experiment. (Bottom) A sequence of images showing different stages of the wall perching from approaching to taking off.

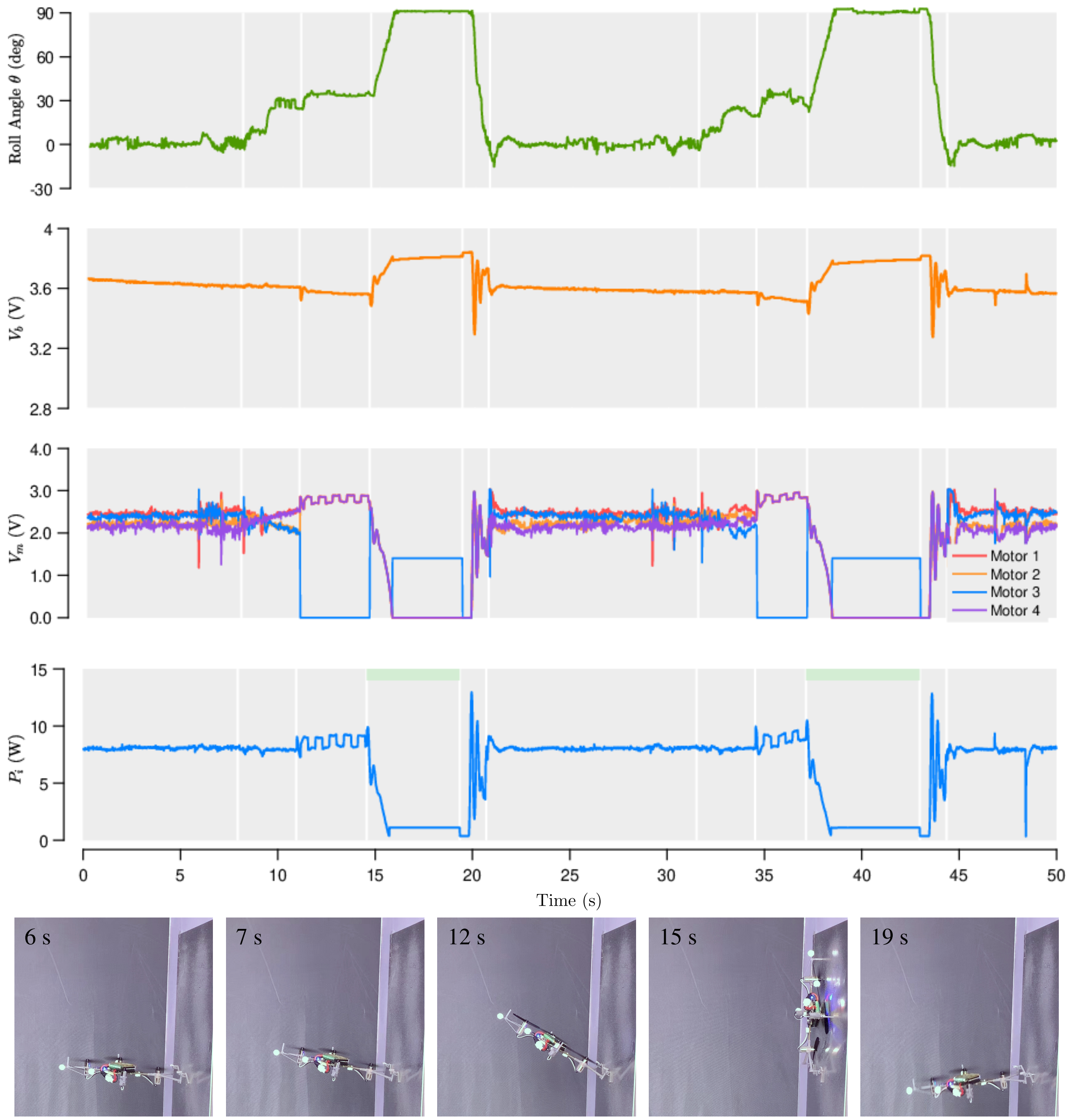

Supplementary Figure 20: Wall perching on wet aluminum. (Top) Plots of flight data taken from the consecutive wall perching experiment. (Bottom) A sequence of images showing different stages of the wall perching from approaching to taking off.

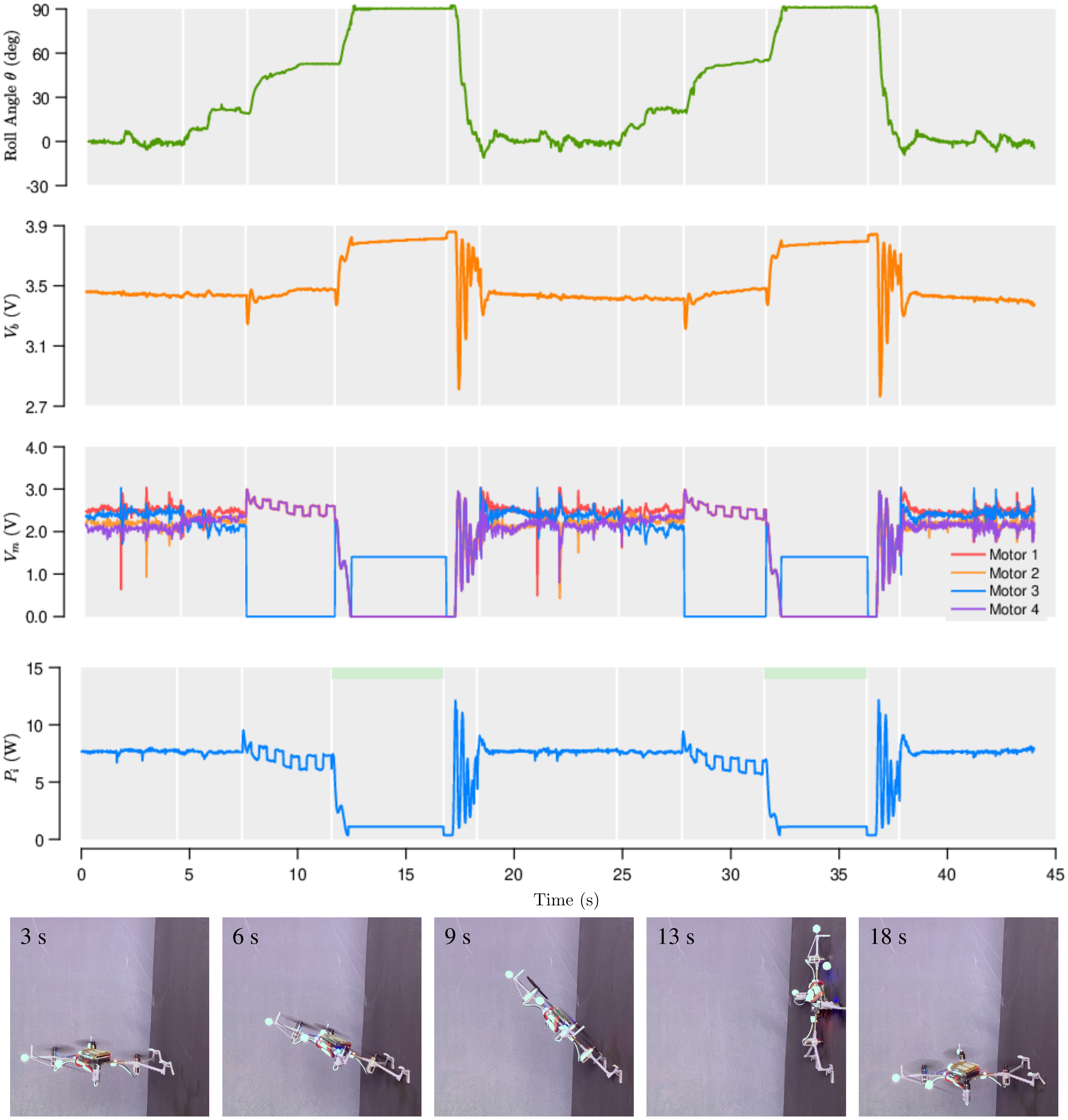

Supplementary Figure 21: Wall perching on dry EVA. (Top) Plots of flight data taken from the consecutive wall perching experiment. (Bottom) A sequence of images showing different stages of the wall perching from approaching to taking off.

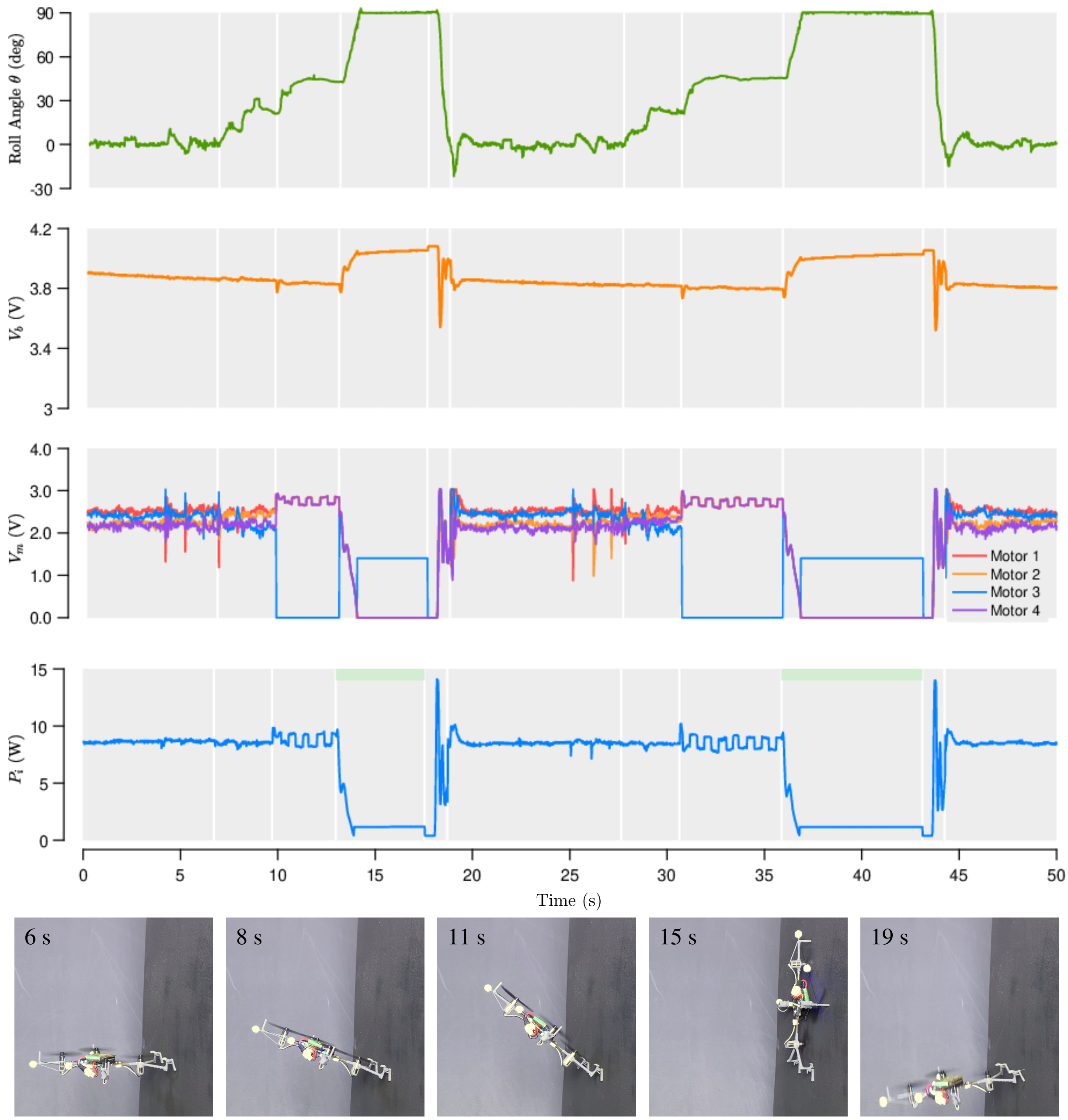

Supplementary Figure 22: Wall perching on wet EVA. (Top) Plots of flight data taken from the consecutive wall perching experiment. (Bottom) A sequence of images showing different stages of the wall perching from approaching to taking off.

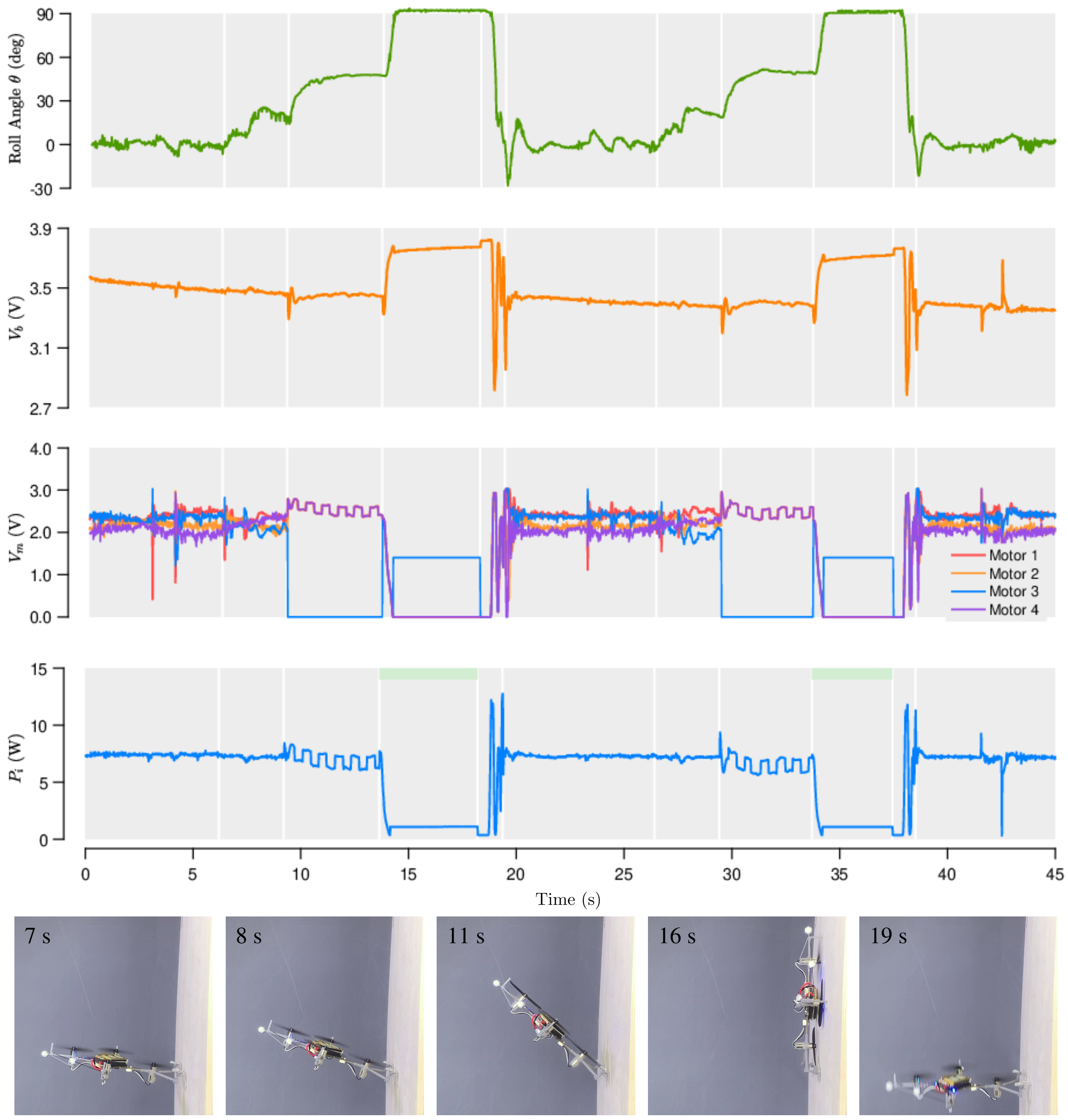

Supplementary Figure 23: Wall perching on dry wood. (Top) Plots of flight data taken from the consecutive wall perching experiment. (Bottom) A sequence of images showing different stages of the wall perching from approaching to taking off.

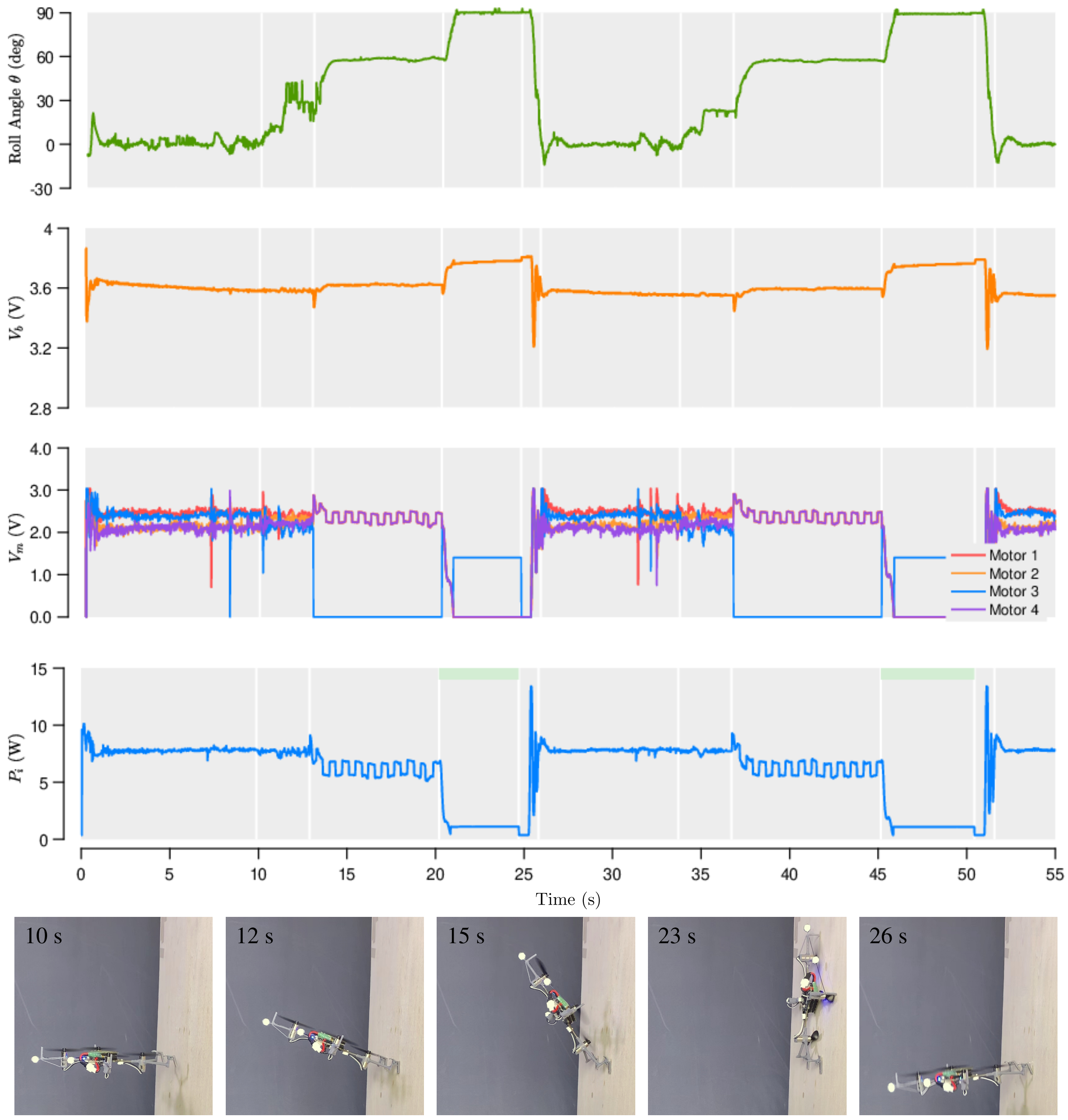

Supplementary Figure 24: Wall perching on wet wood. (Top) Plots of flight data taken from the consecutive wall perching experiment. (Bottom) A sequence of images showing different stages of the wall perching from approaching to taking off.

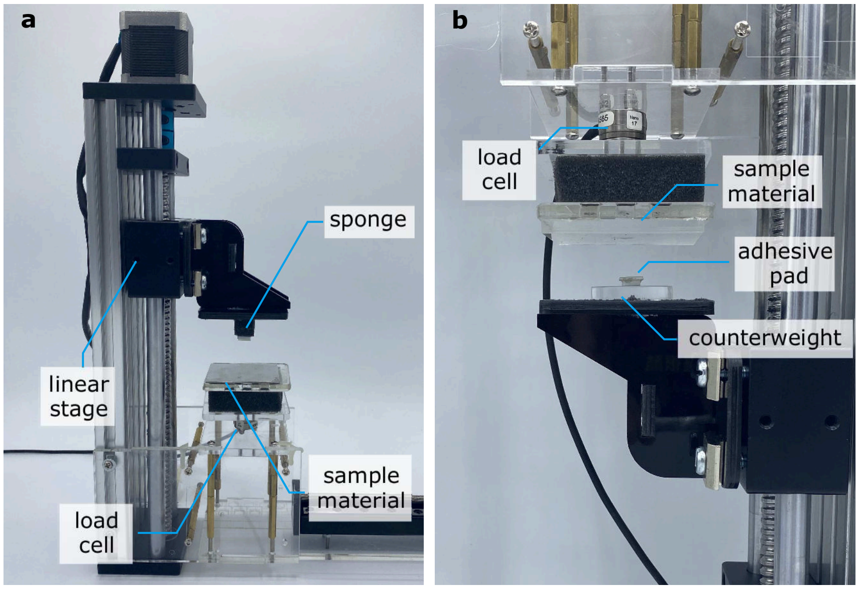

Supplementary Figure 25: Experimental setup for the adhesive characterization. (a) Photograph of the platform compatible with the adhesion pressure tests, the reusability tests, and the creep resistance tests. (b) A closed-up view of the module for the creep resistance tests.

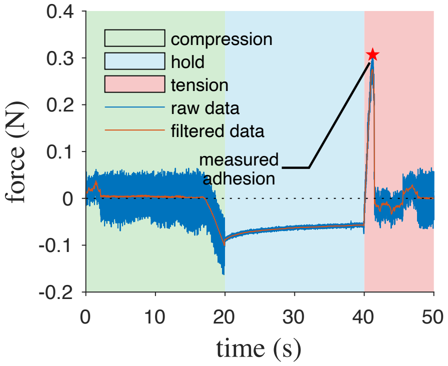

Supplementary Figure 26: An example of the raw measurements of the adhesion pressure test and adhesive reuseability test. Negative force denotes the compression (preload) generated by the stage.

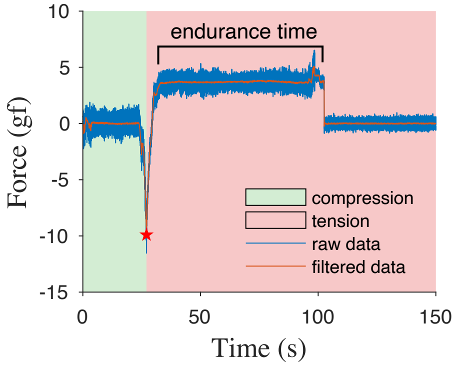

Supplementary Figure 27: An example of the raw measurements of the adhesive creep resistance test. Positive force denotes the pulling force.

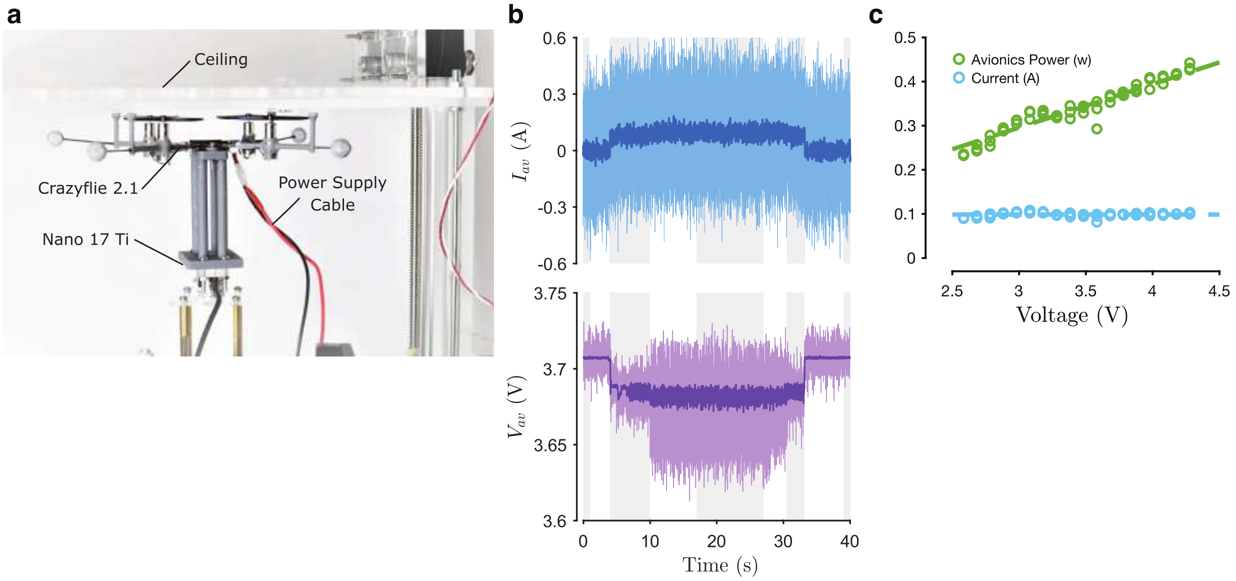

Supplementary Figure 28: Power measurement. (a) Experimental setup with Crazyflie 2.1, ATI Nano 17, and the ceiling. (b) Example results from the avionics power measurements. The raw data is shown in light colors and the filtered results are displayed in dark colors. (c) The consolidated results showing the current and power consumed by the idle flight control board.

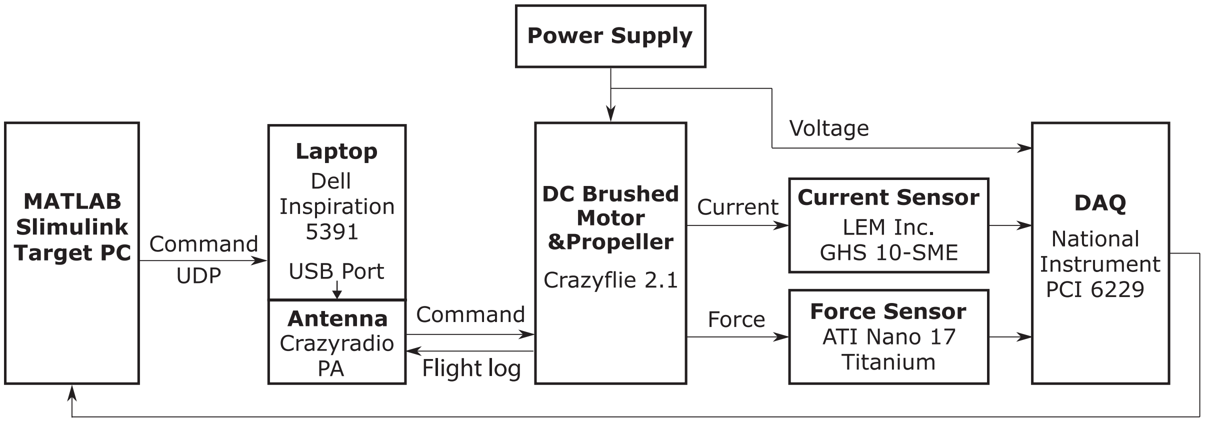

Supplementary Figure 29: Schematic diagram of the experimental setup for power measurements.

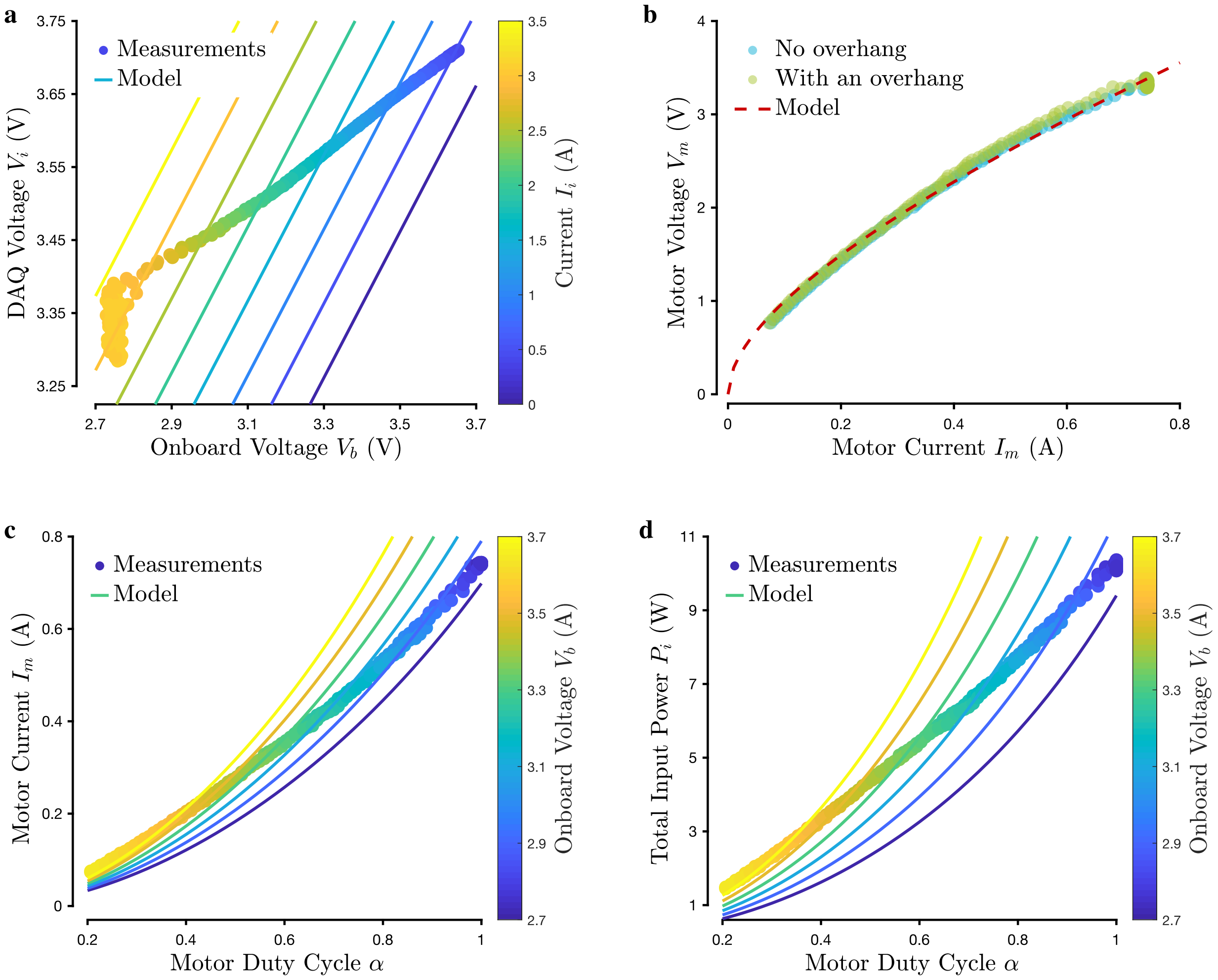

Supplementary Figure 30: Measurements and predictions for the thrust and power models. (a) The relationship between voltages measured onboard and from the DAQ. (b) The plot between the motor voltage and current showing no significant differences whether there exists a proximate surface. (c) The current consumed by the motor depends on both the duty cycle command and the onboard voltage. (d) The total power consumed by the robot also depends on both the motor commands and the onboard voltage.

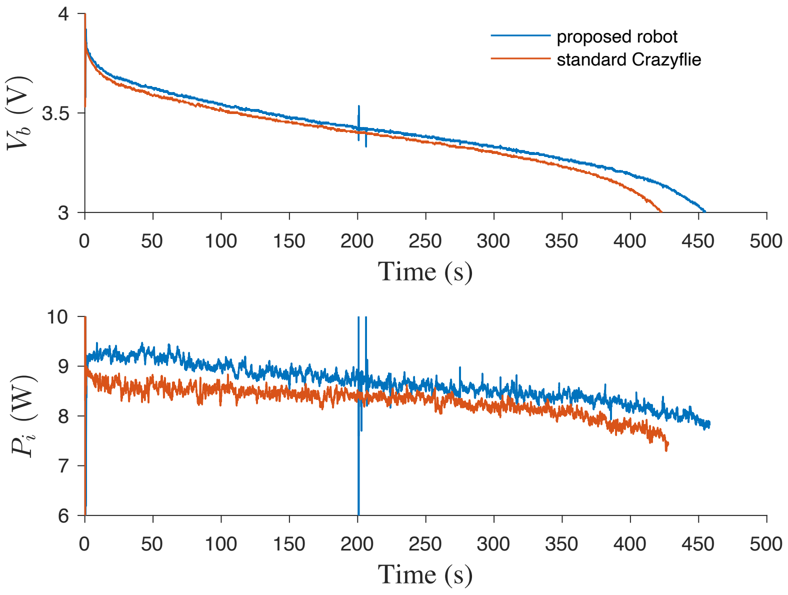

Supplementary Figure 31: Input power and onboard voltage of the proposed robot and original Crazyflie in extended hovering flights. The averaged power consumption in hovering of the proposed robot and the original Crazyflie are 8.6 W and 8.3 W.

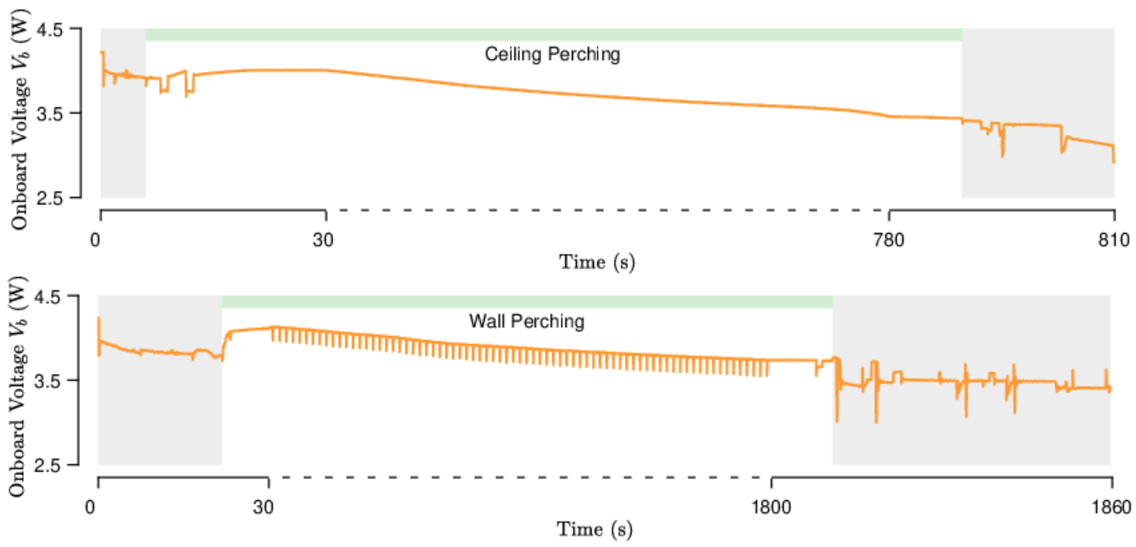

Supplementary Figure 32: Onboard voltage of the robot in extended perching flights. (Top) Ceiling perching. (Bottom) Wall perching.

Table 1: Examples of perching aerial vehicles and their attachment mechanisms

robots from	flight platforms	added weight	total weight	weight ratio	perching surfaces	perching mechanisms: attachments	perching mechanisms: actuators	perching mechanisms: others
Graule et al. [38]	flapping-wing	13.4 mg	97.4 mg	0.14	ceiling	electroadhesion		(without power autonomy)[1]
Gomez-Tamm et al. [18]	flapping-wing	-	450 g	-	branch	claws	SMA springs	(two claws, four fingers each)
Kovac et al. [31]	fixed-wing glider	4.6 g	6 g	0.77	wall	spines	motor	springs
Desbiens et al.[32]	fixed-wing	28 g	400 g	0.07	wall	spines	SMA	elastic linkage
Stewart et al. [23]	fixed-wing	170 g	850 g	0.20	branch	claw/gripper	servo	springs
Estrada et al. [58]	multirotors	-	100 g	-	ground	microspines	servo	(for manipulation)
Zhang et al. [24]	multirotors	10 g	40 g	0.25	branch	compliant grippers,	motor	
Broers and Armanini [25]	multirotors	45 g	294 g	0.15	branch	soft grippers	servo	rubber band
Kirchgeorg and Mintchev [26]	multirotors	40[2] g	400 g	0.10[2]	branch	spines	-	elastic ribbon
Thomas et al. [27]	multirotors	158 g	658 g	0.28	branch	claw	servo	
Roderick et al. [28]	multirotors	250 g	750 g	0.33	branch	claw/gripper	motors	springs, tendon
Doyle et al. [29]	multirotors	478 g	1011 g	0.47	branch	gripping feet	servo	
Hang et al. [30]	multirotors	440 g	1560 g	0.28	branch	gripper	servo	
Nguyen et al. [19]	multirotors	140 g	1766 g	0.08	branch	grapple	motor	(motorized winch)
Popek et al. [20]	multirotors	372 g	2300 g	0.16	branch	gripper	motor	(manipulator)
Mclaren et al. [21]	multirotors	551 g	-	-	branch	robotic hand	servo	tendon, springs
Liu et al. [22]	multirotors	920 g	3800 g	0.24	branch	fingers	motor	(four motorized fingers)
Pope et al. [34]	multirotors	11 g	37 g	0.30	wall	microspines	servo	bow spring (wall climbing)
Daler et al. [33]	multirotors	-	300 g	-	wall	dry adhesive	-	passive self-alignment system
Kalantari et al. [35]	multirotors	-	550 g	-	wall	dry adhesive	servo	springs
Tsukagoshi et al. [36]	multirotors	160 g	1700 g	0.09	wall	suction cups	servo, pumps	
Park et al. [39]	multirotors	3.4 g	20.4 g	0.17	ceiling	electroadhesion		
Jiang [37]	multirotors	15 g	150 g	0.10	wall, ceiling	microspines	servo	springs
**This work**	**multirotors**	**1.1 g**	**32.15**	**0.03**	**wall, ceiling**	**bio-inspired adhesive, proximity effect**	-	

[1]Not included in Figure 1 due to the lack of power autonomy.

[2]Each spine module weighs 5 g. The estimated weight of 40 g assumes the robot has eight spine modules.

Table 2: Robot's physical parameters

Parameter	Description/remark	Magnitude	Unit
**Robot**			
m	total robot's weight	32.15	g
	robot's weight without retroreflective markers	31.00	g
d_{cg}		63	mm
d_{ct}	$r_c + r_p(1/2 - 2\xi_1)$		
	when $\xi_1 = 1/4$	63	mm
	when $\xi_1 = 0$	81	mm
d_{jv}		5	mm
d_{jh}		66	mm
d_{jt}	$r_w + r_p(1 - 2\xi_1 - 2\xi_2)$		
	when $\xi_1, \xi_2 = 1/3$	59	mm
	when $\xi_1, \xi_2 = 0$	106	mm
d_{wj}		6	mm
d_{wg}	$= d_{jh} + d_{wj}$		
r_c		63	mm
r_w		71	mm
r_p		35	mm
**Adhesive pads**			
l_c	ceiling adhesive pad length	5	mm
w_c	ceiling adhesive pad width	8.6	mm
A_c	ceiling adhesive pad area	43	mm^2
l_w	wall adhesive pad length	12.4	mm
w_w	wall adhesive pad width	5	mm
A_w	wall adhesive pad area	62	mm^2
