# Supplementary material for: Energy efficient perching and takeoff of a miniature rotorcraft
Source: Commun Eng. 2023 Jun 13;2:38. doi: 10.1038/s44172-023-00087-y (PMC10956013; doi:10.1038/s44172-023-00087-y)
Supplement: Supplementary file 3 — Description of Additional Supplementary Files [file 44172_2023_87_MOESM3_ESM.pdf]

Description of Additional Supplementary Files

File name: Supplementary Movie 1

Description: Overview

File name: Supplementary Movie 2

Description: Multi-material ceiling perching flights

File name: Supplementary Movie 3

Description: Multi-material wall perching flights

File name: Supplementary Movie 4

Description: Extended perching on ceiling and wall Movie

File name: Supplementary Movie 5

Description: Consecutive ceiling and wall perchings

File name: Supplementary Movie 6

Description: Perchings with only onboard feedback
